# Renal TRPM3 Channels Regulate Blood Pressure via Tubuloglomerular Feedback and Plasma Volume Control

**DOI:** 10.1161/HYPERTENSIONAHA.125.25790

**Published:** 2025-10-01

**Authors:** Jorge Rojo-Mencia, Lucía Alonso Carbajo, Marycarmen Arévalo-Martínez, Lucía Benito-Salamanca, Karel Talavera, M. Teresa Pérez-García, José Ramón López López, Pilar Cidad

**Affiliations:** 1 Departamento de Bioquímica y Biología Molecular y Fisiología, Universidad de Valladolid, and Unidad de Excelencia, Instituto de Biomedicina y Genética Molecular (IBGM), CSIC, Valladolid, Spain (J.R.-M., L.A.C., M.A.-M., L.B.-S., M.T.P.-G., J.R.L.L., P.C.).; 2 Department of Cellular and Molecular Medicine, Laboratory of Ion Channel Research, KU Leuven, Belgium (L.A.C., K.T.).; 3 Instituto de Investigación Biosanitaria de Valladolid (IBioVALL), Valladolid, Spain (M.T.P.-G., J.R.L.L., P.C.).

**Keywords:** angiotensin II, blood pressure, hypertension, ion channels, kidney

## Abstract

**BACKGROUND:**

TRPM3 is a nonselective cation channel activated by heat, osmotic pressure, and neurosteroids. It is highly expressed in sensory neurons, where it integrates thermal, chemical, and inflammatory signals to modulate downstream responses, but is also present in the brain, kidney, and cardiovascular system. This distribution suggests a role in cardiovascular and renal regulation. We hypothesize that TRPM3 channels may play a role in blood pressure (BP) regulation via both vascular and renal mechanisms. Therefore, analysis of the vascular phenotype in *Trpm3*-KO mice can provide insights into the channel’s potential contribution to hypertension development.

**METHODS:**

BP was monitored noninvasively in conscious wild-type and *Trpm3*-KO mice under basal conditions and after oral losartan treatment or angiotensin II infusion via osmotic minipumps. TRPM3 expression in vessels and kidney structures was examined using immunofluorescence microscopy and RNAscope with specific cell markers. Ex vivo renal perfusion and pressure myography were used to evaluate vascular responses.

**RESULTS:**

*Trpm3*-KO mice showed a ≈5% BP reduction and resistance to angiotensin II-induced hypertension. Urine [Na⁺] was 35% to 50% higher, and plasma volume was 20% lower, suggesting a renal origin of the hypotension. TRPM3 was localized at the juxtaglomerular apparatus and distal nephron segments involved in plasma volume regulation. *Trpm3*-KO mice exhibited impaired tubuloglomerular feedback, reducing afferent arteriole constriction and NaCl reabsorption.

**CONCLUSIONS:**

TRPM3 channels contribute to BP regulation by modulating kidney function. Blunted tubuloglomerular feedback in *Trpm3*-KO mice disrupts NaCl reabsorption, leading to hypovolemia and lower BP. Thus, renal TRPM3 channels may serve as promising targets for BP regulation.

**NOVELTY AND RELEVANCE**

**What Is New?**

This study identifies renal TRPM3 channels as key regulators of blood pressure.

Loss of TRPM3 impairs tubuloglomerular feedback, leading to nautriuresis, decreased plasma volume and hypotension.

*Trpm3* knockout mice are hypotensive and protected from angiotensin II-induced hypertension.

**What Is Relevant?**

The findings link TRPM3 channel function to the control of blood pressure through kidney-specific mechanisms.

The study provides evidence that altered sodium handling due to TRPM3 deficiency could prevent hypertension.

**Clinical/Pathophysiological Implications?**

Targeting renal TRPM3 channels may offer novel approaches for managing hypertension by enhancing sodium excretion.

Understanding TRPM3’s role could help develop new treatments for patients resistant to current antihypertensive therapies.

The interplay between vascular and renal signaling pathways is central to blood pressure (BP) homeostasis. However, despite significant progress in hypertension research, the precise molecular determinants of BP variability remain incompletely defined. Among the molecular candidates, transient receptor potential channels have emerged as key regulators of vascular physiology, influencing vascular tone, endothelial function, and cellular calcium dynamics in response to diverse stimuli.^1,2^ Within this family, TRPM3 stands out for its selective expression in tissues relevant to cardiovascular regulation, including sensory neurons, blood vessels, and the kidney.^3–5^ Although its physiological functions are best characterized in sensory systems, the distribution of TRPM3 suggests it may be a promising yet underexplored component of the networks governing BP regulation and hypertension development.

TRPM3 is a Ca^2+^-permeable nonselective cation channel belonging to the melastatin subfamily of transient receptor potential channels.^5^ It is highly expressed in nociceptive neurons, where its activation by noxious heat or by the neurosteroid pregnenolone sulfate (PS) evokes pain,^4^ and in pancreatic β cells, where its activation by PS enhances glucose-induced insulin release.^6,7^ TRPM3-deficient (*Trpm3*-KO) mice exhibit specific deficiencies in their response to noxious heat stimuli, but show normal blood glucose levels, suggesting that glucose homeostasis is not critically affected.^4^

In addition to heat and PS, TRPM3 channels are endogenously activated by other physical (such as the hypotonic mechanical stretch) and chemical stimuli.^8–10^ TRPM3 channels can be activated by intracellular ATP and are modulated by phosphatidylinositol phosphates. The stimulatory effect of cytosolic ATP on TRPM3 reflects activation of phosphatidylinositol kinases, leading to resynthesis of phosphatidylinositol phosphates in the plasma membrane.^11^ Changes in phosphatidylinositol phosphate levels, such as those triggered by receptor-mediated signaling, can strongly influence TRPM3 channel activity.

TRPM3 channels seem to have distinct functions in the vasculature as its activation causes contraction of the mouse aorta,^12^ but dilation of mesenteric arteries.^9^ This is due to the distinct cellular expression patterns in these tissues, that is, TRPM3 is a Ca^2+^ entry pathway in vascular smooth muscle cells (VSMCs) of the aorta, but in mesenteric arteries it is expressed in perivascular nerve endings that release of calcitonin gene-related peptide (CGRP) upon TRPM3 activation, leading to the opening of K^+^ channels and relaxation in VSMCs.^13^ These opposing effects in conduit and resistance arteries raise the question on the role of TRPM3 in vascular function at the systemic level. Answering this is not only relevant for fully understanding the pathophysiological roles of this channel in the vasculature and its possible targeting for the treatment of BP disorders, but also to evaluate the side effects that may be associated with the inhibition of TRPM3 in the treatment of other pathologies linked to gain-of-function TRPM3 mutations.^14–16^ We addressed this issue by investigating the cardiovascular phenotype of the *Trpm3*-KO mice.

We observed that *Trpm3*-KO mice were hypotensive, and their BP values were unaffected by AngII (angiotensin II) infusion via osmotic minipumps. In addition, *Trpm3*-KO mice exhibited increased [Na^+^] in urine, suggesting a renal origin for their decreased BP levels. We hypothesize that TRPM3 channels in the kidney play a critical role in renal blood flow regulation and sodium handling through their expression and function in key nephron structures. For that reason, we explored the expression, location, and functional contribution of TRPM3 channels to renal function and renal blood flow regulation. TRPM3 channels were expressed in multiple renal structures, including macula densa cells within the juxtaglomerular apparatus (JGA). TRPM3 channels modulate tubuloglomerular feedback (TGF) by influencing VSMCs tone in afferent arterioles, thereby contributing to blood volume regulation.^17^ Our data indicate that impaired TGF in *Trpm3*-KO mice reduces afferent arteriole contraction and disrupts NaCl reabsorption. This leads to NaCl loss, decreased blood volume, and ultimately lower BP. We propose that targeting renal TRPM3 channels could be a novel approach for BP regulation through kidney-specific effects.

## Methods

### Data Availability

The data that support the findings of this study are available from the corresponding author upon reasonable request

### Ethical Approval

This study was conducted in accordance with the European Community guidelines for the use and care of experimental animals (Directive 2010/63/EU) and was approved by the KU Leuven Ethical Committee for Laboratory Animals and the Institutional Care and Use Committee of the University of Valladolid.

### Statistical Analysis

Plots and graphs were created with Origin Pro 2025 (OriginLab Corp., Northampton, MA). Data processing and analysis were performed using Microsoft Excel software and statistical analysis was performed using Origin Pro or R-Studio. The combined data in the figures are presented as mean±SD, calculated from multiple experiments. For comparisons between 2 groups with normal distribution, Student *t* test, for paired or unpaired data as required, was used to determine *P* values. Alternatively, Mann-Whitney *U* test was used. For comparisons among several groups, 1-way ANOVA followed by Tukey post hoc test was used in the case of normal distributions and equal variances (Shapiro-Wilk test and Levene or Bartlett test were used to test normality and homogeneity of variances, respectively). Alternatively, a Kruskal-Wallis test for nonparametric data was performed. When analyzing the effects of 2 independent variables and determining if there are interactions a 2-way ANOVA or the equivalent nonparametric Aligned Rank Transform ANOVA were used. A repeated measurements ANOVA was carried out to analyze changes in a single variable measured at different times on the same subject. For dose-response curves *P* values were obtained from the *F* test comparison of the fits between the conditions.

## Results

### TRPM3 Contribution to BP Regulation

**Figure 1.**
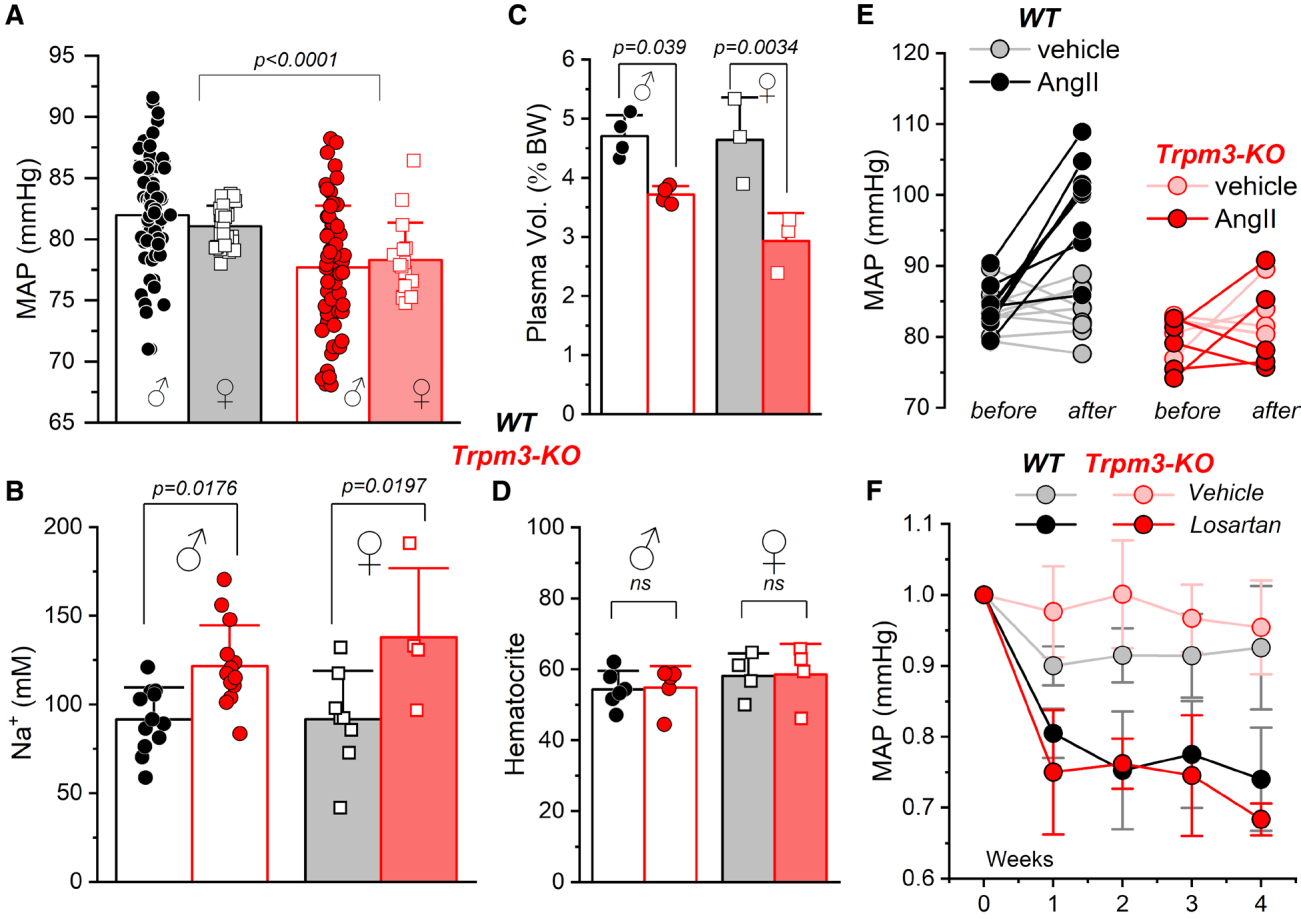
**Phenotypic characterization of *Trpm3*-KO mice. A**, Mean arterial pressure (MAP) in male and female wild-type (WT; N♂=66, N♀=31) and *Trpm3*-KO (N♂=61, N♀=19) mice (Aligned Rank Transform [ART]). ANOVA test indicates significantly lower MAP in *Trpm3*-KO mice, with no effect of sex (*P*=0.92) or strain×sex interaction (**B**) Urinary Na⁺ concentration in WT (N♂=12, N♀=8) and *Trpm3*-KO (N♂=14, N♀=4) mice. Two-way ANOVA followed by Tukey post hoc test. **C**, Total circulating plasma volume normalized to body weight (BW) in male and female WT (N♂=4, N♀=3) and *Trpm3*-KO (N♂=4, N♀=3) mice. Two-way ANOVA followed by Tukey test. **D**, Hematocrit levels (percentage of erythrocyte volume relative to total blood volume from tail vein blood) in male and female WT (N♂=6, N♀=4) and *Trpm3*-KO (N♂=5, N♀=4) mice. Two-way ANOVA followed by Tukey test. **E**, MAP in male WT and *Trpm3*-KO mice before and 7 days after implantation of AngII-releasing minipumps (N WT=9, N KO=5), compared with vehicle-treated controls (N WT=9, N KO=5). A repeated-measures ANOVA followed by post hoc paired *t* tests with Benjamini-Hochberg correction showed a significant increase in blood pressure only in the WT-AngII group (adjusted *P*=0.00017). **F**, MAP in male WT and *Trpm3* KO mice before and after 1, 2, and 3 weeks of losartan treatment (N WT=3, N *Trpm3* KO=3) vs vehicle-treated controls (N WT=4, N *Trpm3* KO=4). MAP values were normalized to show relative changes with respect to *t*=0 for each animal. A repeated-measures ANOVA followed by Tukey test indicates a significant effect of losartan in both WT (*P*=0.0288) and KO (*P*=0.0054) groups, with no strain differences.

The contribution of TRPM3 to BP regulation is difficult to predict, since both vasodilator^13^ and vasoconstrictor effects^12^ upon TRPM3 activation have been reported. To address this question, we determined BP values in wild-type (WT) and *Trpm3*-KO mice. There were no differences between male and female mice, but we found *Trpm3*-KO mice to be slightly but consistently hypotensive compared with the WT (Figure 1A), suggesting a role for TRPM3 channels in BP regulation.

We speculate that the channel could be contributing to BP regulation through long-term mechanisms involving kidney function. In agreement with this hypothesis, we found a significant higher [Na^+^] in urine in *Trpm3*-KO compared with WT (Figure 1B), suggesting renal function alterations in these animals. No sex-dependent differences were observed, as urine [Na^+^] was significantly increased in both male and female *Trpm3*-KO animals. Plasma levels of Na⁺ and other electrolytes, including Ca²⁺, K⁺, and P, showed no significant differences, but there was a significant hypomagnesemia in the *Trpm3*-KO mice (Figure S1). In addition, *Trpm3*-KO mice showed significantly lower circulating blood volume, but no changes in the hematocrit when compared with WT (Figure 1C and 1D; Figure S2). This set of data led us to hypothesize that the hypotensive phenotype of the KO model is due to an abnormal renal function.

To further investigate the association between TRPM3 expression and BP regulation, we used a model of AngII-induced hypertension. BP measurements were carried out in WT and *Trpm3*-KO mice before and after 7 days treatment with control saline (0.9% NaCl) or AngII using implanted osmotic minipumps. AngII infusion induced an overt hypertensive phenotype in WT mice (99±8 mm Hg versus 84±3 mm Hg; *P*=0.00017), but surprisingly, not in *Trpm3*-KO mice, which showed values comparable to those seen in the saline-treated control group (81.26±6 mm Hg versus 81.0±2.5 mm Hg, *P*=0.95; Figure 1E). These results indicate that the absence of TRPM3 confers protection against the development of AngII-induced hypertension, further suggesting an implication of TRPM3 in BP regulation.

The potential differences in the contribution of AngII to BP in control and *Trpm3*-KO mice were explored by treating both strains with the AngII type 1 receptor (AT1R) blocker losartan (estimated dose of 40–60 mg/kg per day) for 4 weeks. Changes in BP were determined weekly (Figure 1F). Chronic treatment with losartan led to a significant BP reduction, without differences between both groups, excluding a different contribution of AT1R in the *Trpm3*-KO mice.

To establish a possible correlation between TRPM3 expression levels in the kidney and BP, we also measured TRPM3 mRNA expression in a well-recognized model of hypertension, normotensive (BPN, BP normal) and hypertensive (BPH, BP high) mouse strains.^17,18^ Interestingly, mRNA expression levels of TRPM3 in kidney were significantly higher in BPH compared with BPN mice (Figure S3). In addition, like *Trpm3*-KO mice, BPN mice showed no significant elevation in BP in response to AngII.^19^ These results indicate that both *Trpm3*-KO mice and mice with low renal expression of TRPM3 channels share a common phenotype of protection against AngII-induced hypertension, pointing toward a role of renal TRPM3 in BP regulation.

### TRPM3 Contribution to Blood Flow Regulation in the Kidney

**Figure 2.**
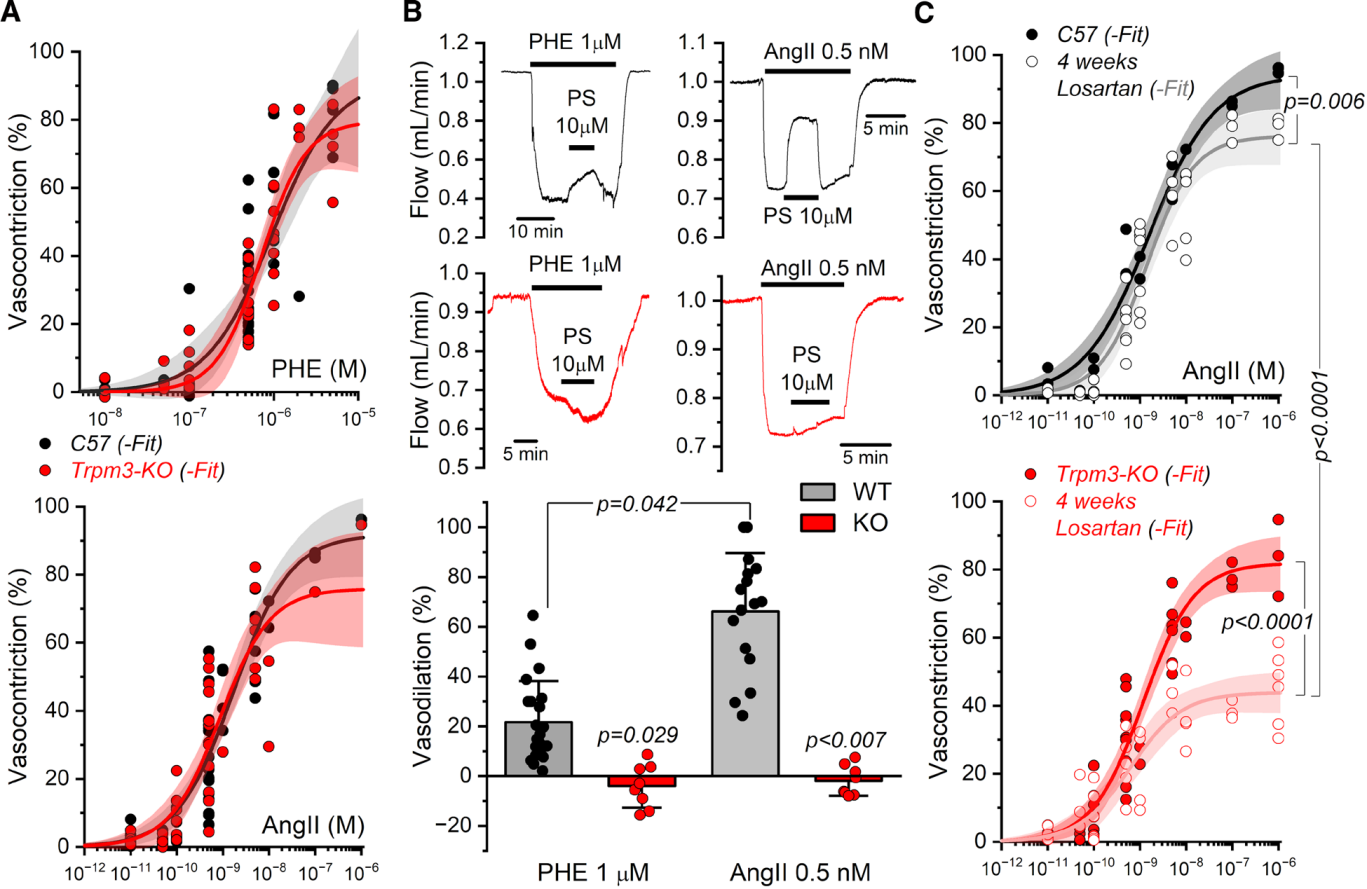
**Renal circulatory reactivity. A**, Dose–response curves showing the vasoconstrictive effect of phenylephrine (**top**) and angiotensin II (**bottom**) in isolated perfused kidneys from wild-type (black) and *Trpm3*-KO (red) mice. Solid lines represent the data fit to a Hill equation, and the shadows represent the confident intervals of the fit. Fit parameters are shown in Table S1. **B**, Representative traces of the effect of pregnenolone sulfate (PS, 10 μM) on renal flow in kidneys prestimulated with phenylephrine (1 μM, left) or angiotensin II (0.5 nmol/L, right). Black traces correspond to wild-type (WT) kidneys and red traces to *Trpm3*-KO kidneys. Bars graphs in the bottom depict the quantification (mean values±SD) of PS-induced vasodilatory responses under phenylephrine (N WT=23, N KO=6) and angiotensin II (N WT=16, N KO=10) conditions. Nonparametric 2-way ANOVA was performed using the Aligned Rank Transform (ART) method. Post hoc pairwise comparisons were conducted using estimated marginal means with Tukey-adjusted *P* values. **C**, Dose-response curves for angiotensin II-induced vasoconstriction in WT (**top**) and *Trpm3*-KO (**bottom**) kidneys after ≥4 weeks of losartan treatment (open symbols) vs control (solid symbols). Solid lines and shadows as in **A**. Fit parameters are shown in Table S2.

To investigate the contribution of TRPM3 channels to renal function, we next examined renal vascular reactivity using a whole-kidney perfusion setup (Figure S4). The renal artery was cannulated, and the kidney was perfused at constant pressure, so changes in flow can be taken as a proxy of changes in the resistance of the entire renal vascular bed. We first determined the renal vasoconstriction responses to phenylephrine and AngII. Fitting the dose-response curves obtained for WT and *Trpm3*-KO kidneys (Figure 2A; Table S1) to Hill equations and comparing the models using an *F* test showed no statistically significant differences. Remarkably, although *Trpm3*-KO mice did not develop hypertension after AngII treatment, their vascular response to AngII-induced constriction was unaffected.

Next, we evaluated the effect of TRPM3 activation on these responses (Figure 2B) by applying 10 µmol/L PS in the presence of either 1 µmol/L phenylephrine or 0.5 nmol/L AngII. In both cases, PS elicited vasodilation, which was significantly larger when AngII was used as vasoconstrictor. Of note, the lack of effect of 10 µmol/L PS in *Trpm3*-KO mouse kidneys (Figure 2B, red traces) shows that the specificity of PS at this concentration as an agonist of TRPM3 holds in the kidney vasculature precontracted with either phenylephrine or AngII, as previously described for phenylephrine-precontracted mouse mesenteric arteries.^13^ The larger responses to PS obtained in the presence of AngII suggest that the contribution of TRPM3 channels to the modulation of blood flow depends on the signaling context in the kidney. In fact, the effect of PS weakly correlates with the amount of vasoconstriction elicited by the prestimulation with phenylephrine or AngII (Figure S5), but the slope of the correlation is larger for AngII. Similarly, we observed that chronic AT1R blockade with losartan reduces the maximal response to acute AngII, and this effect is more pronounced in *Trpm3*-KO mice (Figure 2C; Table S2). This indicates that the chronic blockade of AT1R weakens this signaling pathway to a larger extent in the absence of TRPM3 channels.

Based on prior studies on the expression of TRPM3 in sensory neurons,^4,13,20^ we hypothesized that the TRPM3-dependent vasodilation induced by PS in the renal system could be dependent on the release of CGRP by perivascular nerves. To investigate this, we conducted expression studies in isolated renal arteries, and functional experiments to evaluate renal blood flow modulation by pharmacological agents.

**Figure 3.**
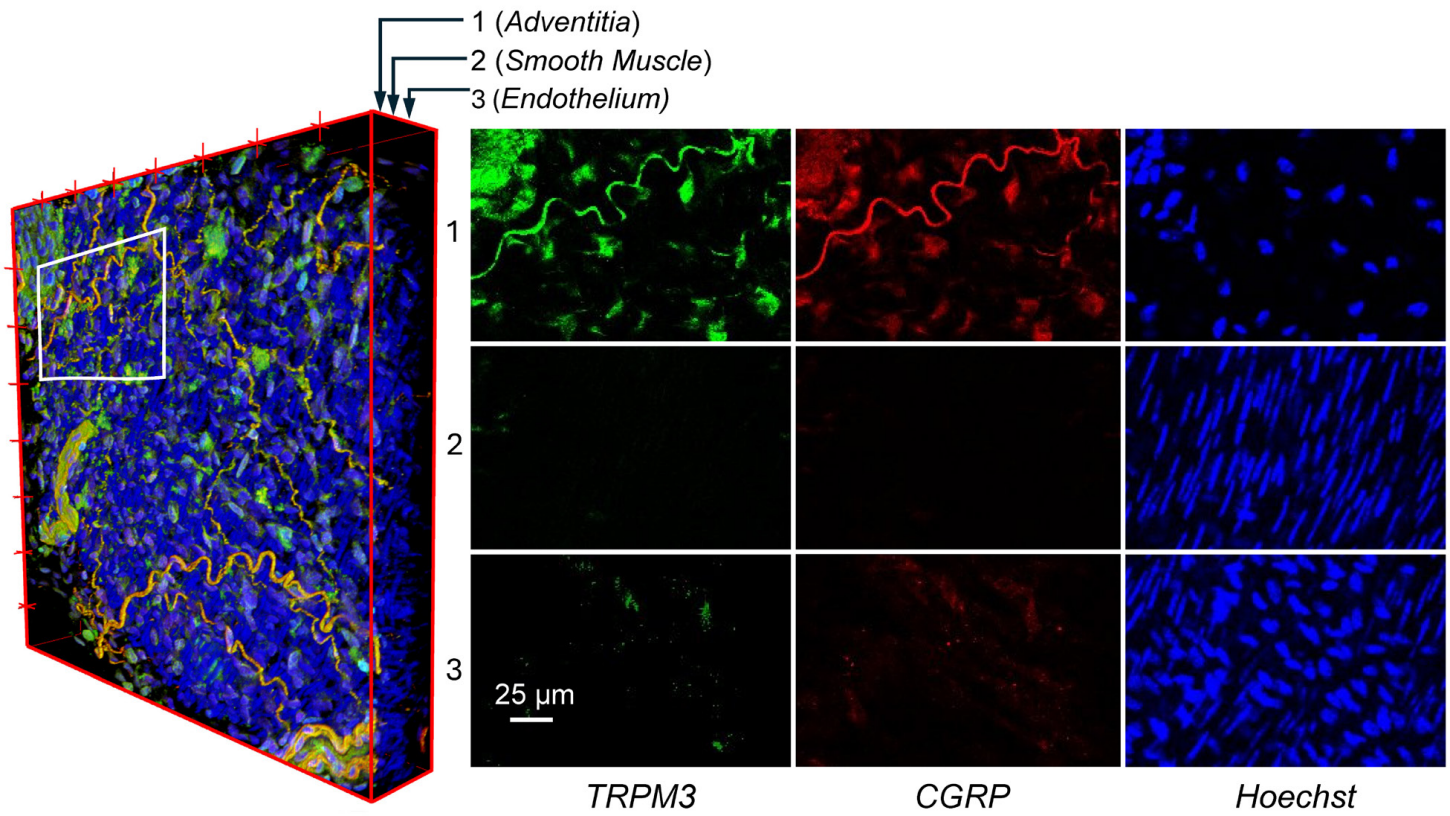
**Localization of TRPM3 in renal artery perivascular sensory nerves.** Left, 3D reconstruction of a longitudinal segment (≈400 µm) of an isolated renal artery from a *Trpm3*-KO using a z-stack of 59 confocal images sectioning all arterial layers. TRPM3 channels are labeled using β-galactosidase staining (green), sensory nerves identified by CGRP immunostaining (red) and nuclei were counterstained with Hoechst (blue). Zoom images corresponding to the labeled white rectangle and from 3 confocal sections at the level of adventitia (1), tunica media (2) and endothelium (3) are shown to the right. Images are representative of 5 fields of independent arterial samples from 5 different animals.

In the absence of reliable anti-TRPM3 antibodies, we took advantage of the fact that *Trpm3*-KO mice have incorporated a β-galactosidase (β-gal) reporter into the reading frame of the *Trpm3* gene.^13^ We performed immunolabelling with an anti-β-gal (14B7) mouse mAb (which detects *E*. *coli* β-galactosidase fusion proteins) and anti-CGRP antibodies in whole renal arteries of *Trpm3*-KO mice to test the colocalization of the transgene product and CGRP using confocal microscopy (Figure 3). β-gal colocalized with CGRP in the perivascular nerves, and both markers were absent in VSMCs and endothelial cells. As in the case of mesenteric arteries,^13^ these result shows that TRPM3 expression in renal arteries is restricted to the perivascular nerves in the adventitial layer.

**Figure 4.**
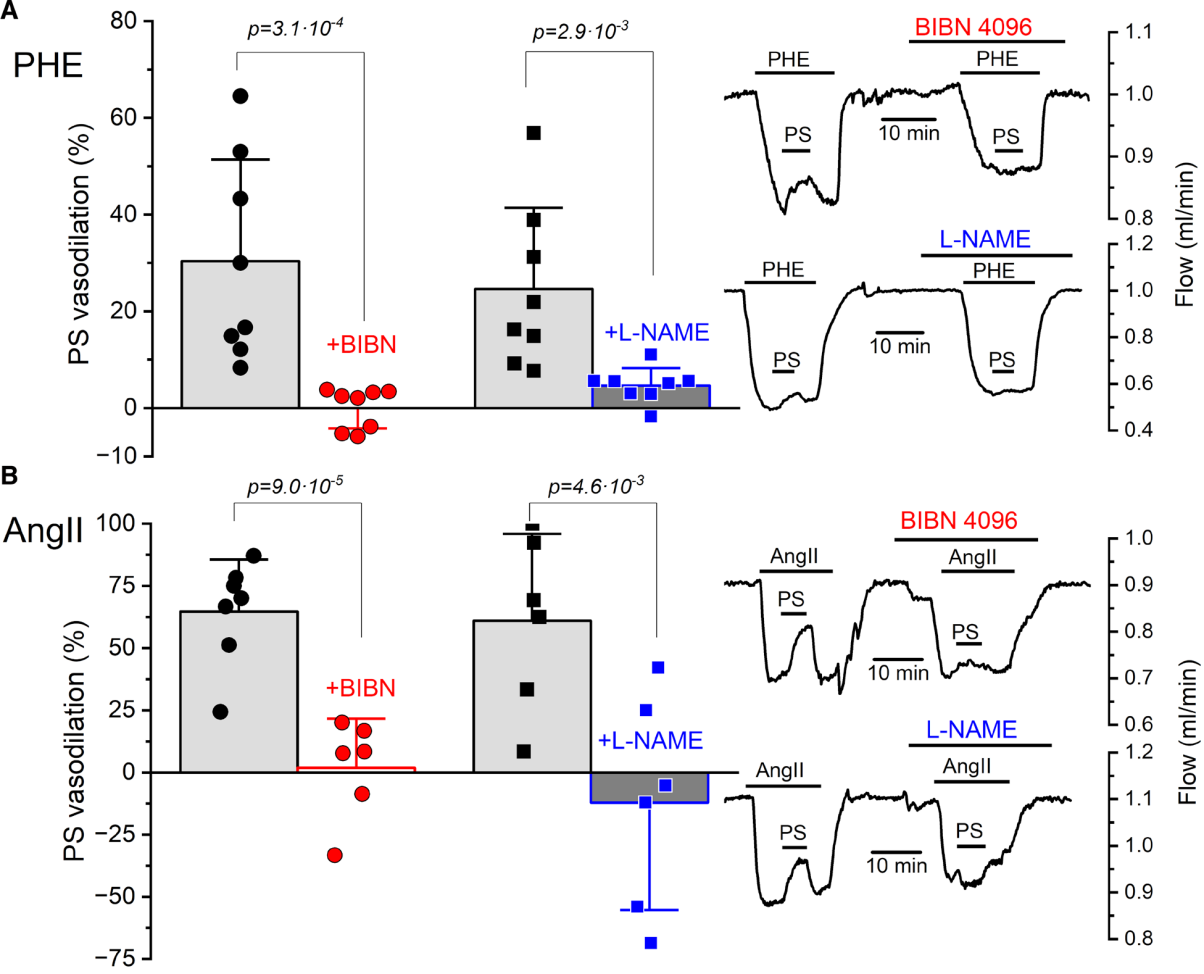
**Effect of TRPM3 activation on renal flow after inhibiting canonical vasodilatory pathways.** Responses to pregnenolone sulfate (PS, 10 μM) during phenylephrine (PHE, 1 μM; **A**) or angiotensin II (AngII, 0.5 nmol/L; **B**) induced vasoconstriction in isolated perfused kidneys from wild-type mice, in the presence or absence of the CGRP receptor blocker BIBN4096 (1 μM) or the NOS blocker L-NAME (100 μM). In each case representative traces are displayed on the right panels, and the average data from 6 to 9 animals in each set of experiments is shown on the bar plots. Statistical significance was evaluated using Student paired *t* test.

Next, we tested the effect of 10 µmol/L PS on renal flow in the presence of the CGRP receptor antagonist BIBN4096 (1 µmol/L). In this condition, PS-induced vasodilation on phenylephrine-contracted arteries was fully prevented (Figure 4A), indicating that the TRPM3-mediated effect of PS depends on CGRP receptor activation. Similar results were obtained with pressure myography of renal arteries (Figure S6). The vasodilating action of CGRP is mediated by the stimulation of CGRP receptors in endothelial cells and VSMCs.^21,22^ Therefore, part of the effect of PS is expected to be mediated by CGRP-induced NO release from endothelial cells. PS-induced vasodilation in phenylephrine-precontracted renal arteries was almost fully abolished in the presence of the NOS blocker L-NAME (100 µmol/L; Figure 4A), suggesting that TRPM3-induced vasodilation is mainly mediated by the endothelium. In contrast to BIBN or L-NAME, 10 µmol/L indomethacin had not significant effects on the PS responses (Figure S7A).

Similar results were observed when BIBN4096 was tested with AngII as the precontractile stimulus (Figure 4B). However, the results were different to those obtained with phenylephrine in the presence of 100 µmol/L L-NAME or 10 µmol/L indomethacin (Figure S7B). In some experiments, the PS-induced vasodilation observed in the absence of blockers unexpectedly shifted to vasoconstriction when applied with the blockers. We conclude that TRPM3 activation in perivascular nerves promotes renal artery vasodilation through CGRP- and NO-dependent pathways. Yet, when these pathways are inhibited, TRPM3 activation can enhance AngII-induced vasoconstriction, suggesting that TRPM3 channels may also be expressed and functionally important at other sites within the kidney beyond perivascular nerves.

### TRPM3 Location in Kidney

TRPM3 labeling with β-gal was attempted in renal parenchyma but failed to produce clear results due to a high autofluorescence that compromised the signal-to-noise ratio. In fact, the strong fluorescence background in kidneys represents a well-recognized difficulty in immunofluorescence assays.^23^ 3,3’-diaminobenzidine-staining of recombinant β-gal allowed detection only in the *Trpm3*-KO kidneys, but with limited sensitivity (see Figure S8).

**Figure 5.**
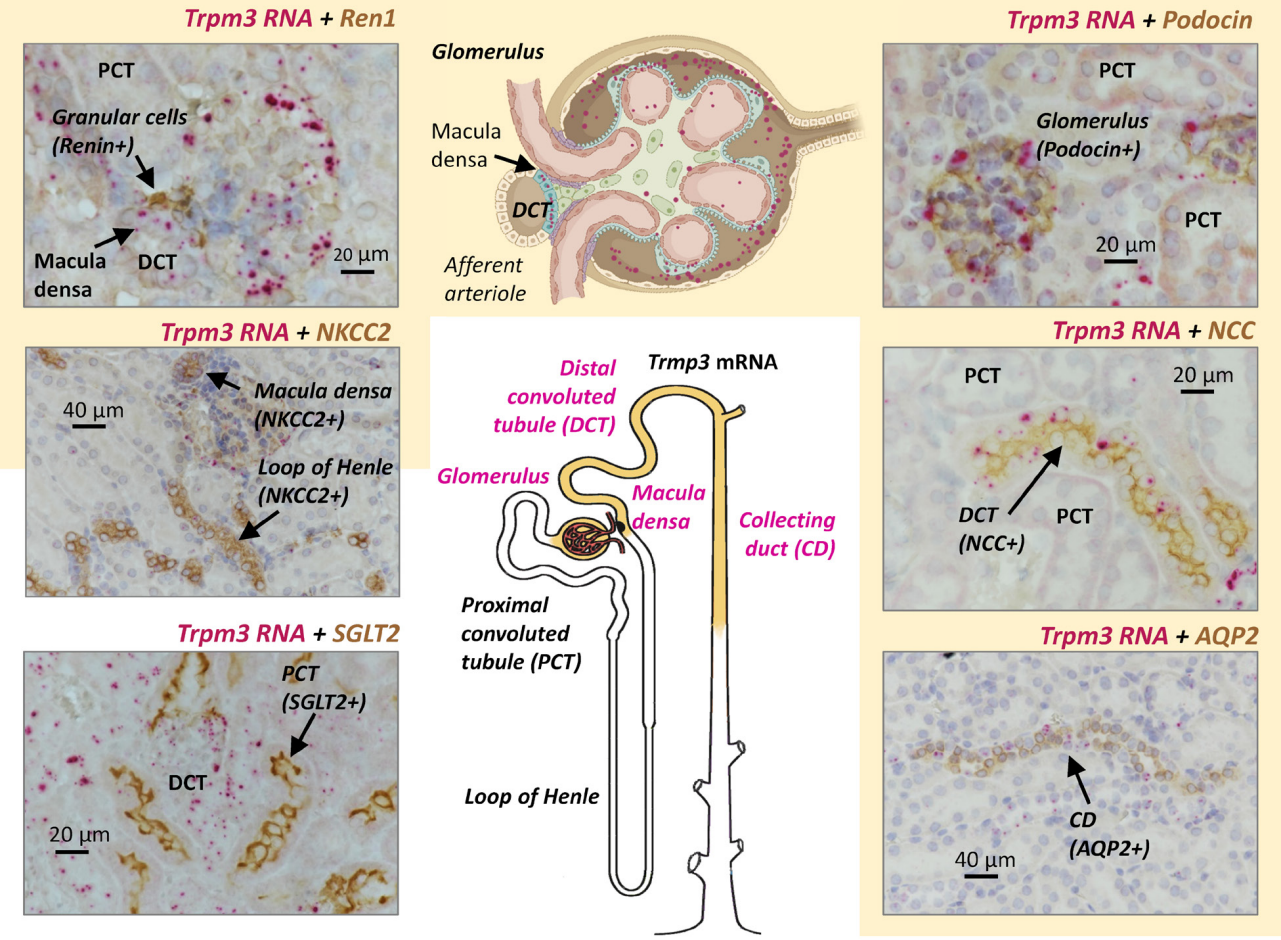
**TRPM3 mRNA localization in nephron segments.** Brightfield images of 5 µm kidney sections from wild-type mice immunohistochemically labeled (brown) using the following markers as indicated in each image: granular cells (Ren1), proximal tubule (SGLT2), Henle loop and macula densa cells (NKCC2), glomerular podocytes (Podocin), distal convoluted tubule (NCC), and collecting duct (AQP2). TRPM3 mRNA expression was detected by RNAscope in situ hybridization (red dots). The nephron regions showing positive TRPM3 RNA signals are highlighted in yellow in the accompanying schematic. Each image is representative of at least 5 fields of 2 to 5 sections from 3 to 5 different wild-type (WT) male animals kidney samples.

To overcome these limitations, we use RNAscope to map TRPM3 expression in murine kidneys (Figure 5). RNAscope is an advanced in situ hybridization technique that allows visualization of individual RNA transcripts with low background. It combines high specificity (due to probe design), high sensitivity (due to signal amplification), and spatial resolution (preserving tissue architecture), making it ideal for mapping the expression of low-abundance genes in complex tissues, as is the case for TRPM3 expression in the kidney.^24^

The results from positive and negative controls for RNAscope are shown in Figure S9. *Trpm3*-KO mice tissues could not be used as negative controls, as the global KO expresses a truncated protein^25^ and hence TRPM3 mRNA expression could also be detected with RNAscope (Figure S9B). Using the TRPM3 probes, we detected positive signals (red dots in Figure 5) only in certain renal structures from WT mice, which were identifiable by their morphological characteristics and confirmed through co-staining with established cellular markers. We observed clear labeling in distal convoluted tubules (DCT, also stained with sodium-chloride cotransporter), intercalated cells (but not principal cells) of collecting duct (CD, colocalizing with aquaporin-2), and glomerular cells positive for podocin. In contrast, TRPM3 mRNA expression was absent from proximal convoluted tubule cells (no overlap with SGLT2 [Na-glucose cotransporter-2 antibody]) or the thick ascending limb of the loop of Henle, where Na/K/Cl cotransporter positive cells lacked TRPM3 signal. However, Na/K/Cl cotransporter is also expressed in the apical membrane of macula densa cells,^24^ where its activity represents the primary mechanism by which macula densa cells detect the tubular salt concentration and activate TGF.^26^ TRPM3 mRNA staining was observed in macula densa cells colocalizing with Na/K/Cl cotransporter but was absent of other cell types of the JGA, as it did not colocalize with renin labeling of granular cells. Finally, we did not detect TRPM3 mRNA colocalization with anti-SM22 or antityrosine hydroxylase, excluding TRPM3 expression in VSMCs and sympathetic nerve endings, respectively (Figure S10). Altogether, the expression of TRPM3 in the kidney shows a preferential distribution in regions where the monitoring of the composition of the tubular fluid (macula densa) and the subsequent fine-tuning of sodium and water reabsorption (DCT and CD) take place. Macula densa cells are part of the JGA, which plays a dual regulatory role: (1) controlling the tone of afferent arteriolar smooth muscle cells via the TGF mechanism and (2) regulating renin secretion. We found no colocalization of TRPM3 mRNA with renin in granular cells, and no changes in renin activity in plasma between WT and *Trpm3*-KO mice (75±11 in WT versus 70±17 ng/mL in *Trpm3*-KO; *P*=0.48). Thus, we hypothesized that TRPM3 channels may contribute to renal function by modulating the TGF mechanism.

### TRPM3 Contribution to Tubuloglomerular Feedback

The TGF mechanism functions at the individual nephron level, where it adjusts afferent arteriolar resistance and glomerular filtration rate (GFR) in response to changes in NaCl concentrations in the early DCT. This maintains fluid delivery to the distal nephron within a specific range and establishes a negative relationship between macula densa NaCl concentration and GFR or capillary pressure.

**Figure 6.**
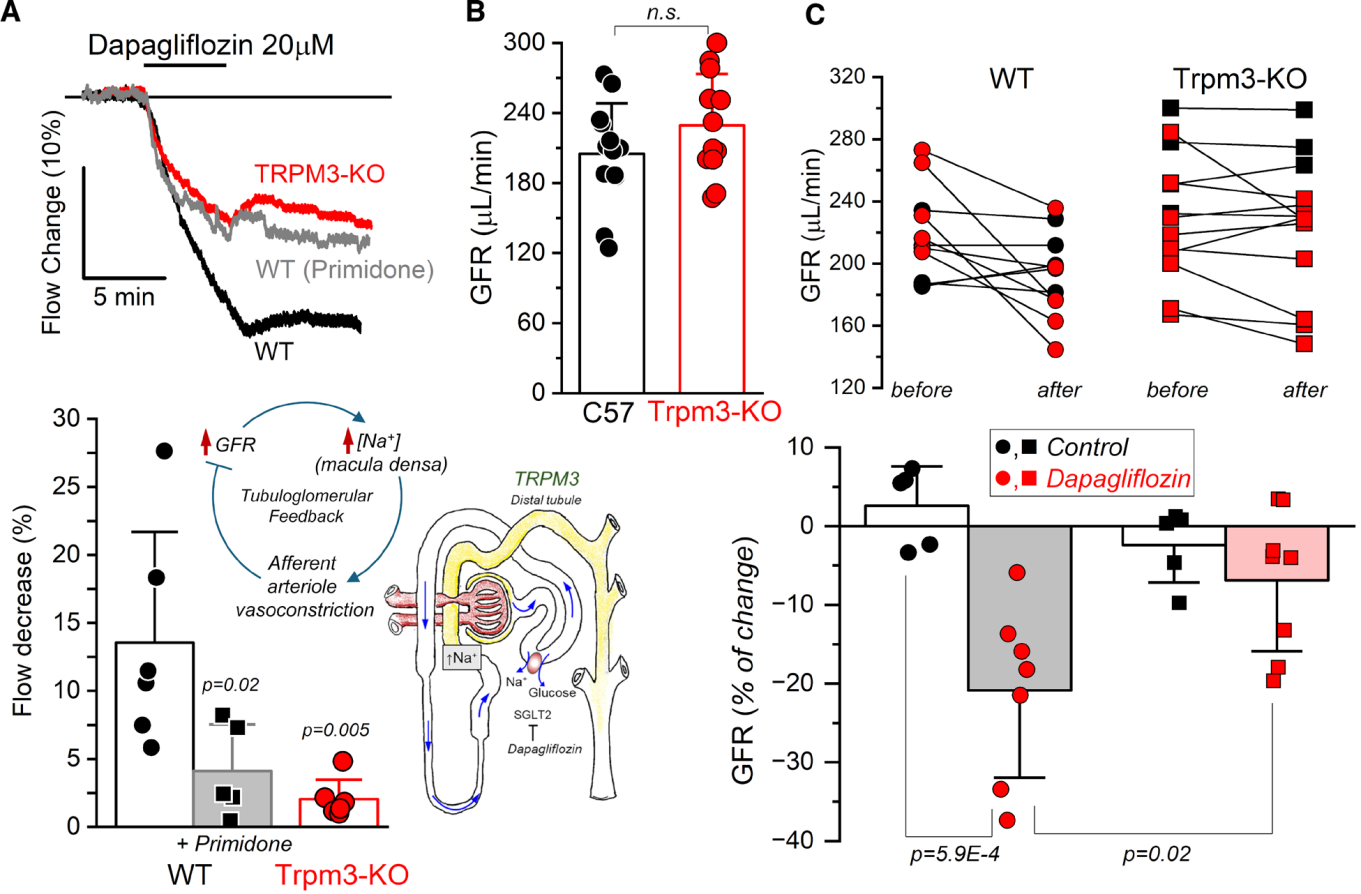
**TRPM3 contributes to the vasoconstriction involved in mediating tubuloglomerular feedback. A**, Effect of 20 μM dapagliflozin (an SGLT2 inhibitor) on renal flow in isolated perfused kidneys from wild-type (n=6), *Trpm3*-KO (n=5), and wild-type mice treated with primidone (a TRPM3 blocker, 10 μM; n=5). Representative traces and average data (mean±SD) are shown. Statistical significance was analyzed by ANOVA followed by Tukey post hoc test. **B**, Glomerular filtration rate (GFR) (µL/min) was determined by measuring fluorescein isothiocyanate-inulin clearance from plasma after retro-orbital bolus injection. The plot shows mean±SD of 12 animals in each group (wild-type [WT] and *Trpm3*-KO). **C**, GFR was determined in the same animals before and after 72 to 96 h treatment with dapagliflozin (25 mg/kg day in drinking water, red symbols) or control (water, black symbols). In the graph in the bottom, changes in GFR after treatment are estimated as % of the GFR before (n=5 control and 7 treated mice in each group). A 2-way ANOVA followed by post hoc Tukey analysis indicated that dapagliflozin significantly reduced GFR in WT mice, but had no significant effect in KO mice. In addition, GFR decrease was significantly smaller in treated KO mice compared with WT.

To investigate whether this mechanism is altered in *Trpm3*-KO mice, we examined renal blood flow responses to dapagliflozin, an SGLT2 inhibitor. SGLT2 inhibition reduces Na^+^ and glucose reabsorption in the proximal tubule, increasing Na^+^ delivery to the DCT. Macula densa cells should detect the increase in Na^+^ and trigger the afferent arteriole vasoconstriction. As expected, Figure 6A shows that 20 µmol/L dapagliflozin reduced renal flow in WT mice, and this effect was significantly smaller in *Trpm3*-KO mice. Furthermore, when we administered the TRPM3 channel blocker primidone (10 μmol/L) to WT animals, the dapagliflozin-induced contraction was significantly reduced, strongly suggesting a role for TRPM3 channels in the vasoconstrictor response responsible for the TGF. To further validate TGF impairment in vivo, we measured GFR using fluorescein isothiocyanate-inulin clearance following retro-orbital bolus injection, both under basal conditions and after 72 to 96 hours of dapagliflozin treatment.^27^ Baseline GFR did not differ between WT and *Trpm3*-KO mice (Figure 6B). However, dapagliflozin induced an initial decline in GFR in WT mice, attributable to hemodynamic changes in the kidney (and specifically, to enhanced TGF, as previously described^28^). Notably, this GFR reduction was significantly blunted in *Trpm3*-KO mice, confirming a compromised TGF response in the absence of TRPM3 channels.

## Discussion

BP regulation involves a dynamic interplay between neurohumoral signaling and mechanical factors that modulate systemic vascular resistance. While several vascular adaptations contribute to hypertension,^29,30^ the leading model, based on Guyton pressure-natriuresis concept,^31^ highlights impaired renal Na^+^ excretion as the main cause of chronic hypertension, regardless of its origin. This impairment leads to volume expansion and increased systemic vascular resistance.^32,33^ Accordingly, the basic mechanism of efficacy for diuretics and dietary sodium restriction in hypertension is to favorably influence sodium balance and homeostasis. However, other antihypertensive agents such as RAAS (renin-angiotensin-aldosterone system) inhibitors, vasodilators, and β-blockers work through a similar mechanism by facilitating pressure-natriuresis.

### Role of TRPM3 in Renal Control of BP

Our characterization of the vascular phenotype of *Trpm3*-KO mice are consistent with a predominant role of renal TRPM3 channels in the control of BP. We propose that the hypotensive phenotype in the *Trpm3*-KO is linked to a decrease plasma volume because of increased natriuresis, pointing to the involvement of renal TRPM3 channels in long-term renal control of BP. In support of the link between renal TRPM3 channels and BP levels, real-time PCR analysis demonstrated a significant 2-fold increase in TRPM3 mRNA expression in kidneys from hypertensive BPH mice compared with normotensive BPN mice (Figure S3). Although the evidence is indirect, we found a correlation between TRPM3 expression and basal BP levels within strains.

Notably, TRPM3 is not expressed in proximal tubule segments, where bulk Na⁺ reabsorption occurs (Figure 5). Hence, its action appears to involve downstream nephron segments. It has been shown that the final adjustments of urinary Na^+^ excretion in the distal nephron are important for volume homeostasis. In this line most of the human mutations that cause Mendelian forms of hypertension or hypotension affect fluid reabsorption in the distal portion of the nephron.^34^ Recent models propose that changes in key Na^+^ transporters leading to impaired Na^+^ excretion serve as the final common pathway by which vascular, neural, and inflammatory factors increase BP.^32^ Given the complexity of BP regulation, it is surprising that all the mutated gene products are in the distal portions of the nephron and act in the same physiological pathway.

### TRPM3 Interaction With Angiotensin II and AT1R

Interestingly, both *Trpm3*-KO and BPN mice^19^ exhibit significant protection against AngII-dependent hypertension, as described previously for kidney proximal tube selective *At1r*-KO.^35^ Thus, although the evidence remains indirect, these observations highlight a potential role for TRPM3 in modulating renal RAAS responses and contributing to systemic BP regulation.

Although the precise molecular origin of elevated BP cannot be determined for most hypertensive patients, RAAS pharmacological inhibition consistently reduces BP, underscoring its critical role. The pressor effects of the RAAS are primarily mediated by AngII acting on AT1Rs, which are expressed in multiple tissues, including the central nervous system, vasculature, heart, adrenal glands, and the kidney.^36^ Using global and kidney-specific *At1r*-KO mice models, it has been shown that at basal conditions, AT1Rs in both kidney and extrarenal tissues make distinctive contributions to BP homeostasis that are virtually equivalent in magnitude and independent of each other.^37^ However, their relative contributions are quite different in response to AngII-induced hypertension, as the absence of AT1R in the kidney alone was sufficient to protect from AngII-dependent hypertension, while the presence of AT1R in the kidney alone in systemic KOs is sufficient to recapitulate the hypertension phenotype.^33,35^

AngII causes hypertension primarily through effects on AT1R in the kidney associated with reduced urinary Na^+^ excretion, independent of actions of the sympathetic nervous system or aldosterone. In fact, abrogation of AT1R signaling only in the proximal tubule-KO is sufficient to lower BP, despite intact vascular responses. Elimination of this pathway reduces proximal fluid reabsorption and alters expression of key Na^+^ transporters, modifying pressure-natriuresis and providing substantial protection against hypertension.^38^ Interestingly, proximal tubule-KO mice also exhibit changes in the expression and function of transporters in downstream nephron segments,^39^ which can be related to the mechanisms involved in the effects of TRPM3 channels, as they are not expressed in the proximal tubule (Figure 5).

### Contribution of TRPM3 Channels to Tubuloglomerular Feedback

Previous single-cell RNAseq data from mouse kidneys show *Trpm3* gene expression along the nephron and in the renal vasculature, with a more abundant expression at CD^24^ (see also https://humphreyslab.com/SingleCell/search.php). Differences with our data are likely due to the low expression levels of TRPM3 channels, which hinder precise localization and limit functional interpretation in high-throughput data sets, in the absence of functional evidence. The selective location of TRPM3 mRNA at the glomeruli, macula densa cells, DCT and CD suggest a role of these channels in TGF and fine-tuning of electrolyte reabsorption. Here, we have tested the hypothesis that the lack of TRPM3 channels at macula densa cells would impair TGF, but there could be other changes in renal function in the KO mice related to other channel locations. As an example, the significant decrease of plasma [Mg^2+^] may be linked to TRPM3 absence in the DCT, a key site for magnesium reabsorption in the kidney. This aligns with clinical findings that dysfunction in DCT proteins like TRPM6 or sodium-chloride cotransporter causes magnesium wasting and hypomagnesemia.^40^ Within each nephro-vascular unit, the tubule returns to the vicinity of its own glomerulus. At this site, the specialized tubular cells (macula densa cells), sense changes in tubular fluid composition and transmit information to the glomerular arterioles to regulate GFR and blood flow. TGF allows changes in the NaCl content of tubular fluid passing the macula densa, leading to a reciprocal change in single nephron GFR. Macula densa cells are salt sensors that generate factors that ultimately exert effects on the afferent arterioles, including VSMCs and granular cells.^41^ Two major regulatory functions are performed by the JGA: The TGF (the afferent arteriolar vasoconstriction induced by high tubular [NaCl], a negative feedback) and the renin release induced by the low tubular [NaCl] (a positive feedback mechanism). These divergent mechanisms allow independent fine-tuning of BP. *Trpm3*-KO mice showed unchanged renin secretion and no TRPM3 expression in renin-producing cells, but demonstrated impaired TGF and decreased blood volume, a possible mechanism underlying their hypotension. SGLT2 blockade with dapagliflozin revealed this deficiency, showing diminished vasoconstrictor response in *Trpm3*-KO kidneys compared with WT kidneys (Figure 6). Moreover, dapagliflozin-induced vasoconstriction in WT kidneys was significantly reduced in the presence of the TRPM3 blocker primidone, and dapagliflozin-induced decline of GFR in vivo was blunted in *Trpm3*-KO mice, confirming TRPM3 channel contribution to this effect.

Regarding the mechanism(s) involved, TRPM3 channels are osmoresponsive, and can be activated by the hyperosmolarity associated to NaCl increase in the renal fluid,^42^ contributing to the depolarization and the increase in [Ca^2+^]_i_ in the macula densa cells, which is necessary and sufficient to induce TGF^43^ triggering the release of signaling molecules acting on adjacent mesangial and arteriolar VSMCs. Although the exact molecular identity of these mediators remains incompletely understood due to the complex structure of the JGA, a key step is the regulated release of ATP from macula densa cells, which may directly activate P2 purinergic receptors on neighboring cells or be enzymatically converted into adenosine, both of which play crucial roles in modulating TGF.^44^ Further research to clarify the roles of adenosine and P2 receptors in the impaired TGF observed *Trpm3*-KO mice is warranted.

The absence of TRPM3 expression in the small intrarenal vessels seems to exclude direct vascular tone modulation as a mechanism for BP regulation. In addition, as in the case of mesenteric arteries, the expression of TRPM3 in the large renal arteries seems to be confined to perivascular CGRP-expressing sensory nerve endings, where its activation induces vasodilation, although the interplay between these nerves and VSMCs and endothelial cells via paracrine signaling is complex and deserves further investigation. We show that PS (10 µmol/L) induced vasodilation in the presence of phenylephrine or AngII. This vasodilatory effect was completely abolished by either BIBN4096 (a CGRP antagonist) or L-NAME (a NOS blocker). These findings suggest that TRPM3 contributes to renal blood flow regulation through a CGRP- and NO-dependent pathway, while its nephron-specific expression implicates it in TGF mechanisms, potentially explaining the hypotensive phenotype observed in *Trpm3*-KO mice.

Activation of TRPM3 by PS in perivascular nerves elicits renal artery vasodilation through CGRP- and NO-dependent mechanisms. When these pathways are inhibited, however, TRPM3 activation appears to potentiate AngII-induced vasoconstriction (Figure 4). This paradoxical outcome may be attributable to TRPM3 activity within the nephron, although the contribution of alternative vasoconstrictor targets previously identified for PS, such as BK channels, cannot be excluded.^45^

### Sex-Dependent TRPM3 Activity

Recent studies investigating the role of TRPM3 channel activation in vasodilation of human coronary and middle meningeal arteries revealed sex-dependent differences. Specifically, PS-induced greater vasodilatory responses in arteries from females compared with males, which correlates with significantly higher TRPM3 channel expression in female vessels.^46^ Similarly, TRPM3 activation triggers more pronounced nociceptive sensory firing in the meninges of female mice than in males, suggesting that pain processing in female migraine patients may differ from that in males. Together, these neuronal and vascular TRPM3-related mechanisms may help explaining the higher prevalence and severity of migraine attacks in females.^47^

In contrast to these findings, our study did not detect sex-related differences in the contribution of TRPM3 channels to BP or volume regulation. This may be because hypotension in our *Trpm3*-KO mice stems primarily from TRPM3 loss in the kidney rather than from effects of perivascular nerve TRPM3 channels. Indeed, if vascular TRPM3 channels were the main BP regulator, we would expect *Trpm3*-KO mice to develop hypertension, since TRPM3 activation in blood vessels promotes vasodilation.^13^ This observation should be interpreted with caution, as sex differences were assessed in only a few experiments. With no in vivo differences observed, sexes were combined later, limiting detection of subtle effects.

### Study Limitations

The use of a global *Trpm3*-KO represents a significant limitation to the study, as extrarenal neural (altered sympathetic nervous system activity) or humoral changes (such as increased circulating natriuretic peptides) in the global KO could be contributing to the renal phenotype. While we cannot fully exclude systemic influences, the absence of a detectable reduction in renin activity in *Trpm3*-KO mice, as it would be expected with increased ANP secretion or decreased renal sympathetic drive,^48^ makes these explanations less likely, as well as the fact that no changes in sympathetic vasoconstriction were observed in the KO vessels.^13^

Also, backcrossing of WT and *Trpm3*-KO mice every 8 to 10 generations likely reduced genetic drift, but the possibility that some observed differences may be influenced by residual genetic background effects cannot be fully excluded.

Finally, while we present several indirect experimental results suggesting that impaired TGF (resulting in increased urinary [Na^+^] and decreased blood volume) contributes to hypotension and to the reduced response to AngII in *Trpm3*-KO mice, direct demonstration is still lacking. Future studies using metabolic cage urine collections under controlled dietary conditions will be necessary to confirm these findings.

### Perspectives

The evidence here presented points to TRPM3 channels playing a significant role in BP regulation, primarily through renal mechanisms rather than direct vascular effects. In larger renal vessels, TRPM3 appears confined to perivascular sensory nerve endings where it mediates vasodilation through CGRP- and NO-dependent pathways.^13^ However, hypotension in *Trpm3*-KO mice appears to result from impaired TGF, which may lead to increased urinary [Na⁺]. Although other factors like changes in urine concentration could also contribute, this remains a plausible mechanism leading to reduced circulating blood volume, as supported by the attenuated effect of SGLT2 inhibitors on vascular resistance in *Trpm3*-KO kidneys and the blunted early decline in GFR following dapagliflozin treatment in vivo.

The selective expression of TRPM3 in glomeruli, macula densa cells, DCT and CD (but not in the intrarenal vasculature itself), supports its role in renal salt sensing rather than direct vascular tone modulation. The correlation between renal TRPM3 expression and baseline BP levels, alongside the protection against AngII-induced hypertension in *Trpm3*-KO mice, strongly suggests that TRPM3 represents a promising therapeutic target for hypertension management through its involvement in the final common pathway of renal Na^+^ excretion that regulates BP homeostasis. Further studies aimed at disclosing the molecular and cellular processes affected by the absence of TRPM3 channels will provide insight into the underlying mechanisms.

## Article Information

### Acknowledgments

The authors thank Esperanza Alonso for excellent technical assistance and Daniel Naharro and Miguel San José at the Laboratory of Instrumental Techniques of the University of Valladolid for their help with spectroscopy analyses.

### Sources of Funding

This work was supported by grants PID2020-118517RB-I00 and PID2023-146750OB-I00 from the Spanish Agencia Estatal de Investigación (MICIU/AEI/10.13039/501100011033), VA172P20 from the Junta de Castilla y León (JCyL) and G0C6815N from the Flemish Research Foundation (FWO to K. Talavera). J. Rojo-Mencia had a JCyL predoctoral contract and MAM a postdoctoral Margarita Salas training grant from the Spanish Government.

### Disclosures

None.

## Supplementary Material


