# Supplementary material for: Renal TRPM3 Channels Regulate Blood Pressure via Tubuloglomerular Feedback and Plasma Volume Control
Source: Hypertension. 2025 Oct 1;82(12):2085–97. doi: 10.1161/HYPERTENSIONAHA.125.25790 (PMC12626544; doi:10.1161/HYPERTENSIONAHA.125.25790)
Supplement: Supplementary file 1 [file hyp-82-2085-s001.pdf]

# Supplemental material

## Renal TRPM3 Channels Regulate Blood Pressure via Tubuloglomerular Feedback and Plasma Volume Control

Jorge Rojo-Mencia[1], Lucía Alonso Carbajo[1,2], Marycarmen Arévalo-Martínez[1], Lucía Benito-Salamanca[1], Karel Talavera[2], M. Teresa Pérez-García[1], José Ramón López López[1*] and Pilar Cidad[1*]

[1]Departamento de Bioquímica y Biología Molecular y Fisiología, Universidad de Valladolid, and Unidad de Excelencia, Instituto de Biología y Genética Molecular (IBGM), CSIC, Valladolid, Spain.

[2]Department of Cellular and Molecular Medicine, Laboratory of Ion Channel Research, KU Leuven.

*José Ramón López-López and Pilar Cidad are co-senior authors.

CORRESPONDING AUTHOR:

José Ramón López López
jrlopez@uva.es
Departamento de Bioquímica y Biología Molecular y Fisiología, Universidad de Valladolid, and Unidad de Excelencia, Instituto de Biología y Genética Molecular (IBGM), CSIC, Valladolid, Spain.

# Methods

The data that support the findings of this study are available from the corresponding author upon reasonable request.

### Animals and Ethical Approval

Global *Trpm3-KO* mice were generated from C57BL/6J donor mice at KU Leuven as previously described, using homologous recombination [1,2]. C57BL/6J (WT) mice were obtained from Charles River. Colonies of both *Trpm3-KO* and WT mice were housed in the animal facility of the School of Medicine of Valladolid, under temperature-controlled conditions (21 °C) and with unlimited access to food and drinking. *Trpm3-KO* and WT mice were mated every 8-10 generations to regularly refresh genetic backgrounds to minimize genetic drift and maintain results reproducibility. Normotensive BPN/3J and hypertensive BPH/2J mice were obtained from Jackson Laboratories (Bar Harbor, ME, USA) and maintained with inbred crossings [3,4]. All protocols were in accordance with the European Community guidelines for the use and care of experimental animals (Directive 2010/63/EU) and approved by the KU Leuven Ethical Committee Laboratory Animals (ECD) and the Institutional Care and Use Committee of the University of Valladolid.

### In Vivo Procedures

Blood pressure (BP) was monitored using the CODA setup (Kent Scientific Corp. Torrintgon, CT, USA) following manufacturer´s instructions as previously described [3]. Mice were placed in restraining holders and acclimated on a heated platform at 32°C to 35°C for 15 minutes prior to data collection. All measurements were conducted in awake animals at the same time each day to minimize stress and ensure consistency. Each session included 40 cycles of an inflation phase up to 250 mmHg occlusion pressure, followed by a 15-second deflation phase. A volume of 15 µL was used as the minimum tail volume for data collection. The initial 5 cycles of each session were considered training and excluded from the analysis. A minimum of 5 sessions were performed on consecutive days, but data from the first 2 was omitted from final measurements. BP was determined as the average of all values for each animal.

For $Na^+$ determinations, urine was collected from awake mice at three consecutive days by massaging the abdominal area to stimulate micturition. Samples were stored at 4ºC before processing.

[$Na^+$] in urine samples were determined using a 725-ES inductively coupled plasma optical emission spectrometer (ICP-OES). Aliquots of 10 µl of urine sample were diluted to a final volume of 10 ml with 5% $HNO_3$ prepared in Milli-Q water. Calibration curves were constructed with at least eight points, from a stock solution of NaCl (1000 mg/l, ICP standard, Scharlab). A blank and one calibration sample was run at least every 15 samples.

For the determination of plasma electrolytes, 30 µl of sample was diluted with 3 ml of 65% $HNO_3$ and subjected to two heating steps using an UltraWAVE digester (Milestone). After digestion, the samples were diluted to a final volume of 10 ml with MilliQ water. Calcium ($Ca^{2+}$) and phosphorus (P) were analyzed using a 725-ES ICP-OES, while sodium ($Na^+$), potassium ($K^+$), and magnesium ($Mg^{2+}$) were

measured by ICP-MS 7800 (inductively coupled plasma mass spectrometry). Calibration curves were prepared from certified reference materials (ISO 9001, ISO 17025, ISO 17034) at a concentration of 100 mg/l in 5% $HNO_3$.

A group of *Trpm3-KO* and WT mice were subjected to chronic treatment with losartan (Santa Cruz Biotechnology). Losartan was dissolved in water and filtered to a final concentration of 0.6 mg/ml, providing an estimated daily dose of 40-60 mg/kg/day (daily water intake was monitored to be 3.5 ml/mouse/day). WT control strain and *Trpm3-KO* mice aged 6 months were equally divided into control or treated groups. In this later group, losartan was administered in drinking water for 4 weeks, changing water every 3 days. BP values were obtained before starting the experiment and once a week during the treatment.

To evaluate renal function, glomerular filtration rate (GFR) was explored in conscious mice using FITC-inulin clearance following previously described protocols [5]. Briefly, FITC-inulin was dissolved in 0,85% ClNa and dialyzed to remove unbound FITC. Dialyzed FITC-inulin (30 µg/g body weight) was bolus-injected retroorbitally under light isoflurane anesthesia, and ≈20µl of blood from the tail were collected 3, 6, 10, 15, 30, 50 and 75 min after injection form awake mice. FITC-inulin concentration (mg/L) in plasma was obtained from fluorescence measured in a Cytation5 spectrophotometer, and the clearance was estimated by fitting the data to a two-exponential decay curve using Origin software, according to the following equation:

$$I = I_1 \cdot exp\left(\frac{t}{\lambda_1}\right) + I_2 \cdot exp\left(\frac{t}{\lambda_2}\right)$$

Where I is [FITC-inulin] in mg/L and t is time in minutes. GFR was calculated from the fitting parameters and the injected FITC-inulin ($I_0$) using the following expression:

$$GFR\left( {}^{L}/_{min} \right) = \frac{I_0\,(mg)}{\left( I_1 \cdot \lambda_1 + I_2 \cdot \lambda_2 \right)}$$

After GFR determination under basal conditions, mice were treated for at least 72h with Dapagliflozin at a dose of 25 mg/kg.day in drinking water. Glucose concentration in urine was determined at 48h, being >110 mg/l in all cases, and the FITC-inulin clearance time course was repeated.

**Surgical Procedures**

Mice were anesthetized by inhalation of isoflurane using a SomnoSuiteR Low-Flow Anesthesia System (Kent Scientific). Depending on the mice strain, isoflurane was administered at a dose of 1%-2%, at a flow rate of 500 ml/min for induction and 60 ml/min for maintenance.

Hypertension was induced by AngII infusion via osmotic minipumps (model 1007D, Alzet). AngII was dissolved in saline solution (0.9% NaCl). Minipumps were placed subcutaneously in the intrascapular area of anesthetized animals to deliver AngII at a dose of 800 ng/kg/day. Vehicle groups were given saline solutions (0.9% NaCl). BP was measured daily during at least 3 different days

prior to minipump implantation, and again after 7 days treatment with control saline or AngII.

For most end-point procedures, animals were anesthetized by isoflurane as described and then euthanized by decapitation. Around 1 ml of trunk blood samples were collected in tubes containing ethylenediaminetetraacetic acid (EDTA) and centrifuged at 1500 g for 15 min at 4 °C. The plasma was stored at −80 °C for subsequent renin activity assays.

Following euthanasia, abdominal cavity was carefully opened by midline incision, and the intestines were displaced laterally to expose the kidneys. Both kidneys were excised along with a segment of the abdominal aorta and immediately transferred to a Sylgard®-coated plate containing ice-cold (4 °C) aerated (95% $O_2$ and 5% $CO_2$) solution containing (in mM): NaCl 120, KCl 4.2, $MgCl_2 \cdot 6H_2O$ 1.2, $NaCHO_3$ 25, $KH_2PO_4$ 0.6, glucose 11, $CaCl_2$ 0.01, pH = 7.4 (adjusted with NaOH). Dissection and cleaning of perirenal fat and surrounding connective tissue were performed under a stereomicroscope.

For circulating volume determinations 0.1 ml of Evans blue (1µg/µl in PBS) was injected in the left ventricle of anesthetized mice and after 3 min blood was collected in EDTA containing tubes form right ventricle and animals were killed with isoflurane overdose. Samples were centrifuged as above to separate plasma, and Evans blue concentration was determined from absorbance at 620 nm. Hematocrit was determined in awake mice from tail artery blood using heparinized micro-hematocrit capillary tubes. In a separate set of experiments, hematocrit values and red blood cell (RBC) counts were measured from blood collected via cardiac puncture from the right ventricle. Blood samples were drawn into EDTA tubes, diluted 1:1000 with PBS solution, and RBC counts were performed using a Neubauer chamber.

**Immunofluorescence microscopy**

Immunofluorescence on whole arteries was carried out following previously described protocols [2]. Briefly, intact renal arteries were fixed with methanol-acetone (1:1) overnight at -20 °C, permeabilized in PBTx (PBS, 0.25% Triton X-100), and blocked with PBTx containing 2% goat serum for 1 h. Arteries were first incubated overnight at 4 °C with monoclonal rabbit anti-calcitonin gene-related peptide (CGRP; Cat# 14959, Cell Signaling, 1:200) following 2 h incubation with the secondary antibody polyclonal Alexa Fluor™ Plus 594 goat anti-rabbit IgG (H+L) 1:500 (Cat# A11037, Invitrogen). Samples were fixed again with methanol-acetone (1:1) 20 min at -20 °C and then a second incubation with monoclonal mouse anti-β-gal (14B7, Cat# 2372T, Cell Signalling, 1:200) was performed overnight at 4 °C, followed by incubation with polyclonal Alexa Fluor™ Plus 488 goat anti-rabbit IgG (H+L)1:500 (Cat# A32731, Invitrogen) and nuclei counter-stainer Hoescht (1:3000) for at least 2 h at room temperature. All antibodies' incubations were performed in blocking solution. Finally, the arteries were flat-mounted on glass slides using mounting solution Fluoromount-G (Invitrogen).

Confocal images were acquired with the 40X oil-immersion objective (HCX PL APO) of a LEICA SP5 confocal microscope (Leica Microsystems) using LAS software. Series of z-stack images from the adventitial to the endothelial layer of the artery, were acquired by sequential excitation with a white laser at 488 nm

and 594 nm, and with a UV laser at 405 nm for nuclear staining. Images were analyzed using ImageJ processing software.

**X-Gal staining of whole kidneys**

To visualize β-galactosidase activity in renal tissue, whole-mount X-gal staining was performed on freshly isolated mouse kidneys. The renal artery was cannulated to perfuse the kidneys with phosphate-buffered saline (PBS), followed by fixation with fresh paraformaldehyde solution. After washes with PBS containing 1 mM $MgCl_2$, kidneys were perfused with X-gal staining solution (PBS supplemented with 2 mM $MgCl_2$, 20 mM potassium ferrocyanide, 20 mM potassium ferricyanide, and 1 mg/mL X-gal diluted 1:40 from a 40 mg/mL stock solution in DMSO, filtered prior to use). All components were added slowly while heating the solution gently to avoid precipitation, and the pH was carefully adjusted to 7.3. Perfusion was maintained for approximately 15 minutes, or until staining solution exited the renal vein. Organs were then incubated overnight at 37 °C in the same staining solution to allow enzymatic reaction and chromogenic development, rinsed in distilled water for 15 minutes and post-fixed in 4% formaldehyde for 16–24 hours at 4 °C and processed for paraffin embedding and histological analysis.

**RNAscope In Situ Hybridization**

Isolated kidneys were fixed in neutral buffered formalin (NBF) 10% for 2 h and embedded in paraffin with a Tissue Processor SPT-120 (Leica Biosystems). Samples were cut in 5 µm thickness slices on a rotary microtome HM 340 E (Cat# 905190, EprediaTM) and mounted in SuperfrostTM Plus Adhesion Microscope Slided (Cat# J1800AMNZ, EprediaTM).

RNAscope in situ hybridization was performed using the RNAscope® 2.5 HD Detection Kit- RED (Cat. No. 322360, ACD Bio-Techne) following the manufacturer's protocol. Briefly, renal tissue sections of 5 µm were deparaffinized in xylene, followed by dehydration in an ethanol series. Tissue sections were then placed into RNAscope® Target Retrieval buffer at boiling temperature (100 °C to 103°C) in a hot plate for 15 min. After rinsing by deionized water, RNAscope® Protease Plus was added and co-incubated at 40 °C for 30 min in a HybEZ hybridization oven (Advanced Cell Diagnostics, Hayward, CA) before hybridization with specific probes for Mm-Trpm3 mRNA (Cat# 459911), RNAscope® Positive Control Probe Mn-PPIB (Cat# 313911) and RNAscope® Negative Control Probe DapB (Cat# 310043). Hybridization with target probes, preamplification, amplification, and labelled probe and chromogenic detection were performed following manufacturer instructions.

For immunohistochemistry, the samples were incubated overnight, at 4 °C in a wet chamber with primary antibodies to mark specific cell types; polyclonal rabbit anti-podocin IgG (Cat# PA5-79757, ThermoFisher), polyclonal rabbit anti-NKCC2 IgG 1:200 (Cat# PA5-80003, ThermoFisher), polyclonal rabbit anti-AQP2 IgG 1:200 (Cat# PA5-38004, ThermoFisher), polyclonal rabbit anti-SGTL2 IgG 1:100 (Cat# NBP1-92384, Novus Biologicals), polyclonal rabbit anti-NCC IgG 1:250 (Cat# AB3553, Sigma-Aldrich), polyclonal goat anti-renin1 IgG 1: 250 (Cat# AF4277-SP, Biotechne), monoclonal mouse anti-tyrosine hydroxylase IgG 1:200 (Cat# NB300-108, Novus Biologicals), polyclonal rabbit anti-SM22 IgG 1:100 (Cat# ab14106, Abcam) and mouse anti-β-gal (14B7), 1:100 (Cell Signaling).

HRP conjugated secondary antibodies donkey anti-rabbit, goat anti-rabbit and goat anti-mouse were incubated 2 h at room temperature at 1:500. Signal was revealed by DAB reagent at room temperature for 10 to 30 min. Nuclear staining was performed with Gill's haematoxylin for 3 min and samples were mounted with Eukitt mounting medium (Cat# 03989, Sigma-Aldrich).

**Renal Perfusion**

A custom made isolated perfused kidney system was used to conduct ex vivo renal perfusion (Figure 4SA). In this setup, the kidney is placed in a warm bath chamber maintained at 37 °C, with the renal artery cannulated for perfusion. A peristaltic pump (L-100-1s-2, Longer) delivers solutions to the organ at fixed, pre-established pressure levels. This pump is servo-controlled through a pressure transducer system consisting of a PLUGSYS Transducer Amplifier Module (TAM-D) connected to a PLUGSYS Servo Controller Module (SCP) (both from Hugo Sachs Elektronik, Harvard Apparatus). With this configuration, the system monitors the flow required to maintain specific pressure levels, so that changes in resistance of the vessels to agonists and blockers included in the perfusion solution will be recorded as changes in flow. In this way, flow increases represent vasodilation and flow decreases represent vasoconstriction. Perfusion rate is digitized using a MiniDigi 1B (Axon CNS) and recorded with AxoScope 11.2 software.

During the experiments, kidneys were perfused with a solution (Control solution) containing (in mM): NaCl 120, $NaHCO_3$ 25, Hepes 10, KCl 4.2, $KH_2PO_4$ 0.6, $MgCl_2$ 1.2, Dextrose 5, $Cl_2Ca$ 2 and BSA 6 g/dL, (pH = 7.4) at a constant pressure of 80 mmHg.

Kidneys were first perfused with a control solution to establish baseline flow conditions. Subsequently, a vasoconstrictor stimulus (1 µM PHE or 0.5 nM AngII) was applied with the perfusion solution, alone or in combination with other drugs.

The effect of vasoconstrictors ($E_A$) was estimated as percentage of vasoconstriction:

$$E_A = 100 \cdot \left(1 - \frac{F_A}{F_C}\right)$$

where $F_A$ is the renal flow in the presence of the vasoconstrictor and $F_C$ is the renal flow in control solution (Figure S4).

The effects of TRPM3 activation (induced by 10 µM PS application, $E_{PS}$) was measured as percentage of vasodilation relative to the preceding vasoconstrictor effect:

$$E_{PS} = 100 \cdot \frac{\left(F_{PS} - F_A\right)}{\left(F_c - F_A\right)}$$

where $F_{PS}$ is the renal flow in the presence of PS (Figure S4).

This approach allowed to infer changes in renal arteries tone based on the recorded flow through the entire arterial network.

The effects of the blockade of the $Na^+$-glucose co-transporter 2 (SGLT-2) with dapagliflozin (20 µM) were determined at baseline flow conditions, in the absence of vasoconstrictors.

### Myography experiments

Kidneys were extracted as indicated above from male WT mice (8–12 weeks old) and segmentary arteries were isolated under the same conditions.

Isolated arterial segments (2–3 mm in length) were mounted on a pressure myograph system (Danish Myo Technology 110P) by cannulating both ends between two borosilicate glass pipettes and fixed with nylon filaments. The vessel chamber and the artery segments were filled with saline solution containing (in mM): NaCl 120, $CaCl_2$ 2.5, $MgSO_4$ 1.17, KCl 5, $Na_2HPO_4$ 1.18, $NaCHO_3$ 25, EDTA 1 and glucose 10 (pH = 7.4, adjusted with 5% $CO_2$–95% air). The arteries were pressurized to 70 mmHg in physiological saline solution and incubated at 37 °C for at least 15 min to equilibrate before starting the measurements. The external arterial diameter was measured via digital video edge detection (CCD camera) as previously described[3]

Vascular reactivity was assessed by first constricting the arteries with high $K^+$ solution containing (in mM): NaCl 5, $CaCl_2$ 2.5, $MgSO_4$ 1.17, KCl 120, $Na_2HPO_4$ 1.18, $NaCHO_3$ 25, EDTA 1 and glucose 10, pH = 7.4 (adjusted with 5% $CO_2$ and 95% air).

The effect of PS alone or in the presence of the CGRP receptor blocker BIBN4096 (BIBN) was tested in phenylephrine (10 µM) precontracted arteries. At the end of each experiment, vessels were superfused with a solution containing 10 µm nifedipine to determine the maximal vessel diameter upon relaxation. Responses to chemical agents were calculated as % of vasodilation with the formula $100 \cdot (D_X - D_{PHE})/(D_{Nif} - D_{PHE})$, being $D_x$ the diameter in the presence of the drugs.

### RNA isolation and Real-Time qPCR

Total RNA was extracted from renal tissue with TRIzol® Reagent (Invitrogen™) following the manufacturer's instructions and real-time qPCR was performed with Taqman assays (Applied Biosystems) in a Rotor-Gene 3000 instrument using RPL18 (ribosomal L18) as housekeeping. The fold-increase quantification method ($2^{-\Delta\Delta CT}$) was used to determine mRNA levels of TRPM3.

### Plasma renin determinations

Renin activity was measured in plasma samples from WT and *Trpm3-KO* by a renin assay kit (Cat# MAK157, Sigma-Aldrich), according to the manufacturer's instructions. The reaction took place in a 96-wells plate during 30 min and fluorescence intensities (excitation: 540 nm; emission: 590 nm) were measured at 5 min intervals in a Cytation5 spectrophotometer. The slope (change in fluorescence intensity in 1 min) was compared with that of recombinant renin ($R^2$ = 0.9925).

### Reagents

PregS (Pregnenolone sulfate sodium salt, Cat# P162), L-NAME (Nω-Nitro-L-arginine methyl ester hydrochloride, Cat# N5751), Indomethacin (Cat# I7378)

and Dapagliflozin (Cat# SML2804) were purchased from Sigma-Aldrich. The CGRP receptor antagonist BIBN 4096 (Cat# 4561) was obtained from Tocris Bioscience. Primidone (Cat# P7295) was obtained from Supelco.

**Statistical analysis**

Plots and graphs were created with Origin Pro 2025 (Origin-Lab Corp., Northampton, MA, USA). Data processing and analysis was carried out with Microsoft Excel software and statistical analysis was performed using Origin Pro or R-Studio. The combined data in the figures are presented as mean ± SD, calculated from multiple experiments. For comparisons between 2 groups with normal distribution, Student t test, for paired or unpaired data as required, was used to determine P values. Alternatively, Mann-Whitney test was used. For comparisons among several groups, 1-way ANOVA followed by Tukey post hoc test was employed in the case of normal distributions and equal variances (Shapiro–Wilk test and Levene or Bartlett test were used to test normality and homogeneity of variances, respectively). Alternatively, a Kruskal-Wallis test for non-parametric data was performed. When analyzing the effects of two independent variables and determining if there are interactions a two-way ANOVA or the equivalent non-parametric Aligned Rank Transform (ART) ANOVA were used. A repeated measurements ANOVA was carried out to analyze changes in a single variable measured at different times on the same subject. For dose response curves P values were obtained from the F-test comparison of the fits between the conditions.

# References


1. Vriens J, Owsianik G, Hofmann T, Philipp SE, Stab J, Chen X, Benoit M, Xue F, Janssens A, Kerselaers S, et al. TRPM3 Is a Nociceptor Channel Involved in the Detection of Noxious Heat. *Neuron*. 2011;70:482–494. doi:10.1016/j.neuron.2011.02.051
2. Alonso-Carbajo L, Alpizar YA, Startek JB, López-López JR, Pérez-García MT, Talavera K. Activation of the cation channel TRPM3 in perivascular nerves induces vasodilation of resistance arteries. *J Mol Cell Cardiol*. 2019;129. doi:10.1016/j.yjmcc.2019.03.003
3. Daghbouche-Rubio N, Álvarez-Miguel I, Flores VA, Rojo-Mencía J, Navedo M, Nieves-Citrón M, Cidad P, Pérez-García MT, López-López JR. The P2Y6 Receptor as a Potential Keystone in Essential Hypertension. *Function*. 2024;5:1–15. doi: 10.1093/function/zqae045
4. Moreno-Domínguez A, Cidad P, Miguel-Velado E, López-López JRJR, Pérez-García MTT. De novo expression of Kv6.3 contributes to changes in vascular smooth muscle cell excitability in a hypertensive mice strain. *J Physiol*. 2009;587:625–40. doi: 10.1113/jphysiol.2008.165217
5. Qi Z, Whitt I, Mehta A, Jin J, Zhao M, Harris RC, Fogo AB, Breyer MD. Serial determination of glomerular filtration rate in conscious mice using FITC-inulin clearance. *Am J Physiol Renal Physiol*. 2004;286:590–596. doi: 10.1152/ajprenal.00324.2003

# Figures

***Figure S1. Plasma electrolytes concentration in WT and Trpm3-KO mice.***

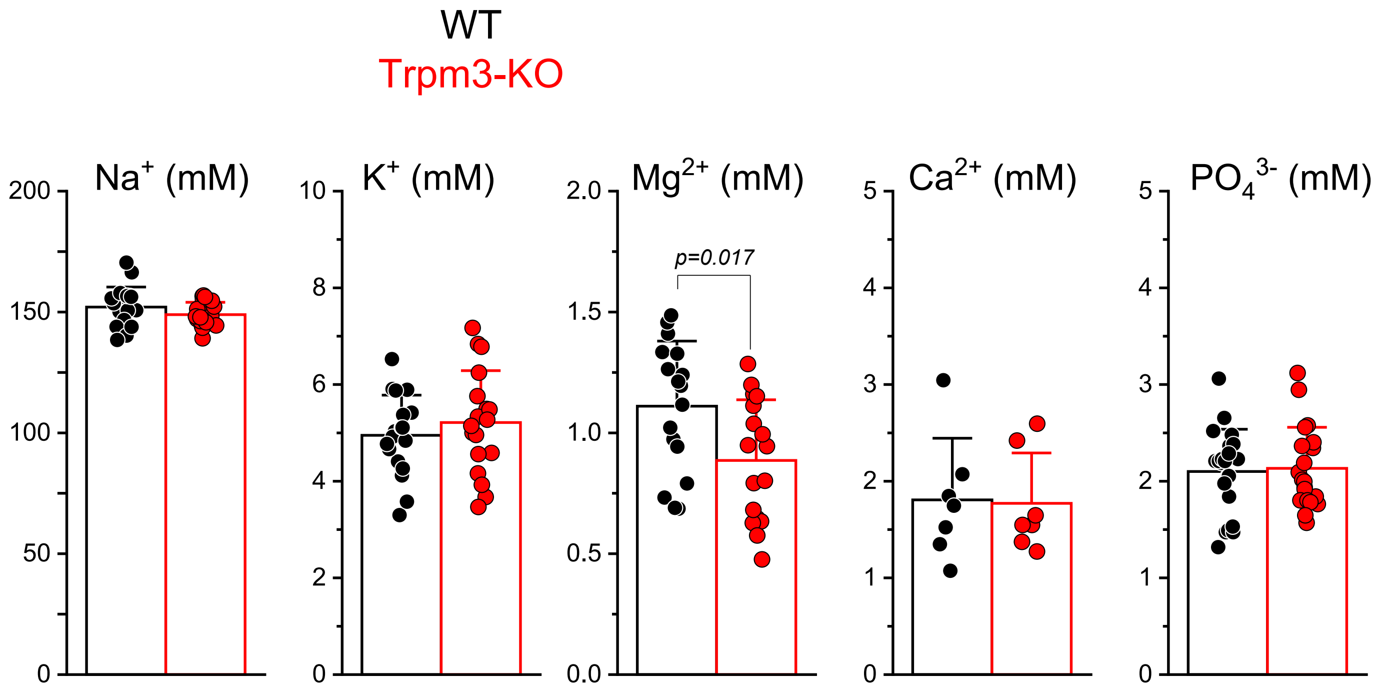


The plots show the plasma concentration (in mM) of the indicated electrolytes in both WT mice (WT, black symbols) and *Trpm3-KO* mice strains (red symbols). $Na^+$, $K^+$ and $Mg^{2+}$ concentrations were determined with inductively coupled plasma mass spectrometry (ICS-MS) and $PO_4^{3-}$ and $Ca^{2+}$ concentrations were obtained with optical emission spectrometry (ICS-OES). Each bar is the Mean ± SD of 9-20 determinations obtained from different mice. A MANOVA (Multivariate Analysis of Variance) indicated a significant multivariate effect of strain on electrolyte levels, (Wilks' Lambda = 0.131, $F(5, 36) = 5.57$, $p < .001$), and following this univariate ANOVAs revealed that only $Mg^{2+}$ levels differed significantly between groups, $F(1, 36) = 6.31$, $p = .017$, indicating that the combined electrolyte profiles differ by strain, and the difference is specifically driven by $Mg^{2+}$ levels.

***Figure S2. Hematocrit and RBC count from right ventricle blood of WT and Trpm3-KO mice.***

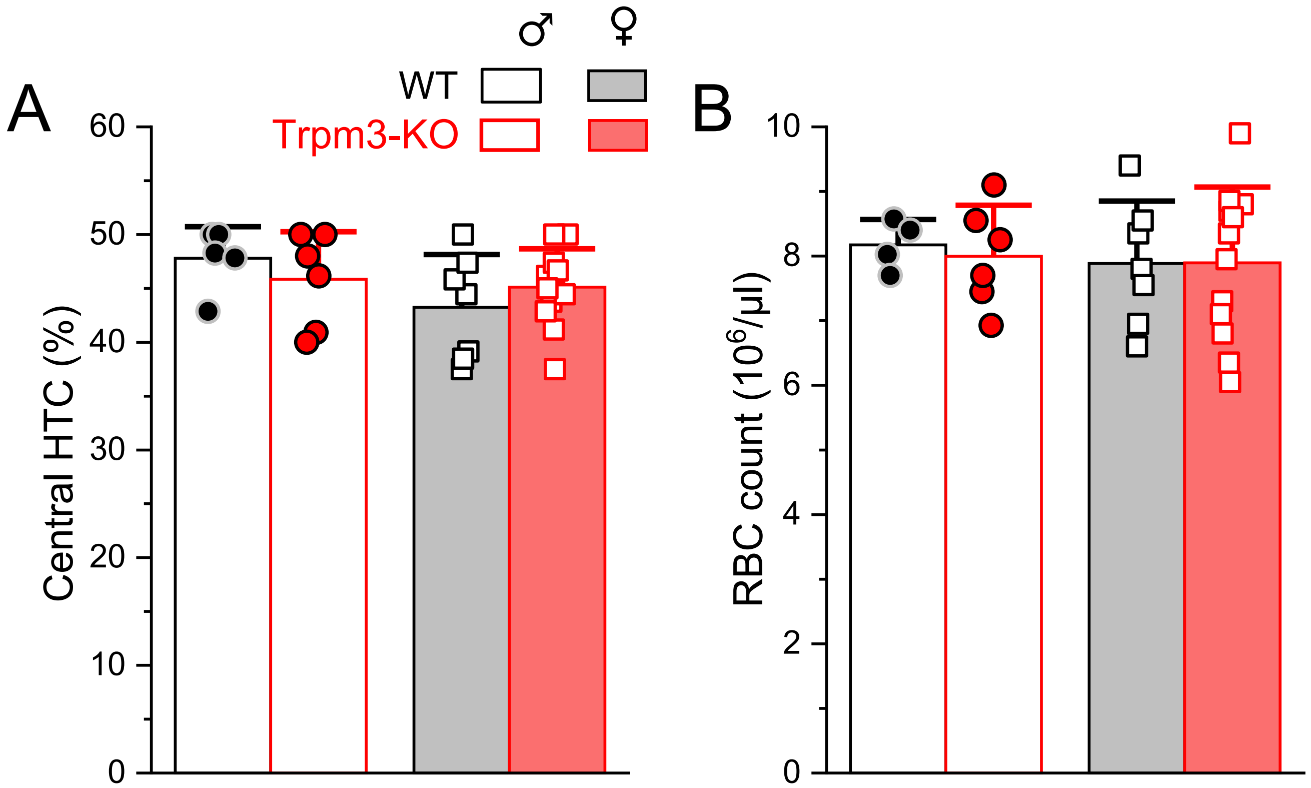


(A) Hematocrit levels (percentage of erythrocyte volume relative to total blood volume) in male and female WT (N♂ = 5, N♀ = 7) and *Trmp3*-KO (N♂ = 6, N♀ = 12) mice. (B) RBCs count was performed in the same samples. In all cases, blood samples were obtained from cardiac puncture. No significant differences in hematocrit or cell count were observed between strains and/or sexes. Differences between these hematocrit values obtained from cardiac puncture and those from tail vein samples (Figure 1D) can be explained by capillary stasis and local hemodynamic factors, which cause RBCs to accumulate in peripheral vessels. Additionally, the practice of 'milking' the tail during sampling results in increased local temperature and an inflammatory response, increasing vascular permeability and promoting fluid extravasation, which leads to higher hematocrit values, as previously described (i.e.,10.1007/PL00000229).

***Figure S3. TRPM3 quantification on a hypertension model.***

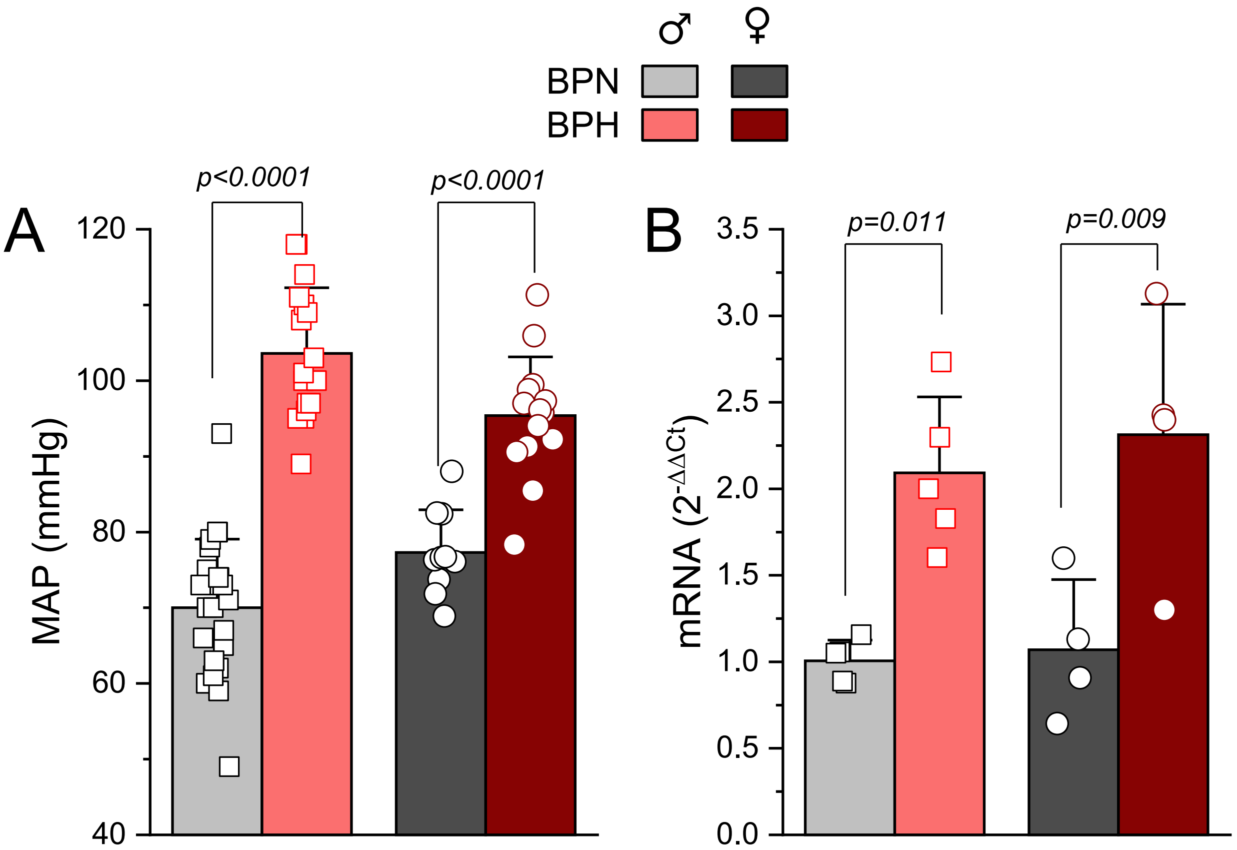


(A) Mean arterial pressure values in *BP normal* (BPN) and *BP high* (BPH) mice, in both males (N BPN = 24, N BPH = 17) and females (N BPN = 10, N BPH = 15). (B) TRPM3 mRNA expression in renal samples from BPH mice (N ♂ = 5, N ♀ = 4) compared to BPN controls (N ♂ = 5, N ♀ = 4), quantified by qPCR and expressed as fold change using the $2^{-\Delta\Delta Ct}$ method. Bars represent mean values ± SD. Statistical significance was tested with a Two-way ANOVA followed by post hoc Tukey´s test.

***Figure S4. Custom-made setup for isolated renal perfusion experiments.***

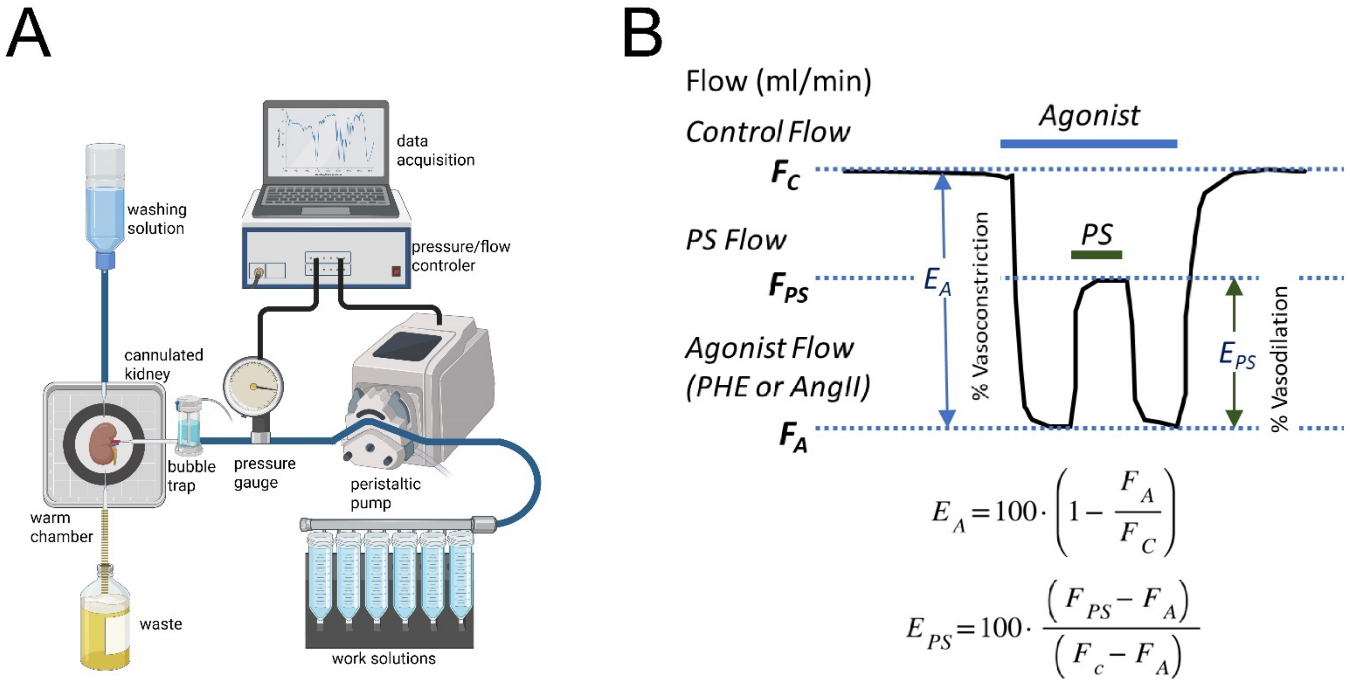


(A) Renal perfusion system consists of a peristaltic pump (Longer L-100-1s-2) delivering solution at controlled pressure to a cannulated kidney placed in a 37 °C bath chamber. Pressure is regulated via a feedback loop between a pressure transducer and a servo-controller module (TAM-D and SCP, Hugo Sachs Elektronik). Changes in vascular resistance to vasoactive agents are detected as variations in flow, which is digitized (MiniDigi 1B, Axon CNS) and recorded using AxoScope software. (B) Scheme of the analysis of the effect of vasoconstrictors (PHE or AngII, $E_A$) as %Vasoconstriction and the effect of PS ($E_{PS}$) as %Vasodilation.

***Figure S5. Correlation the effect of PS with the level of preconstriction obtained with PHE or AngII.***

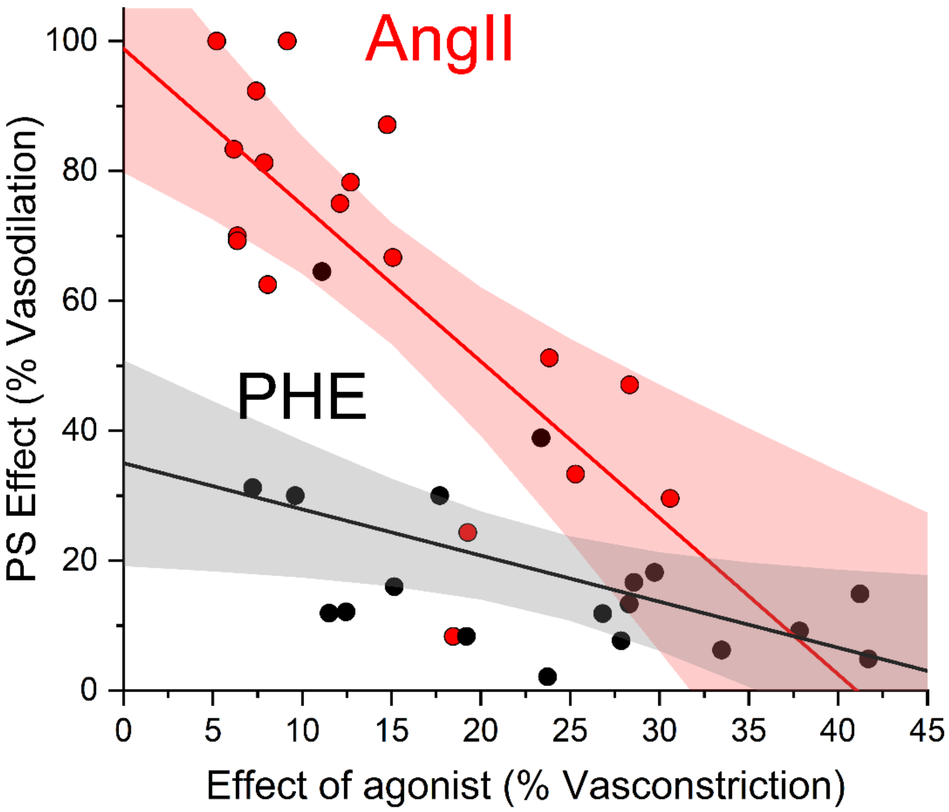


The vasodilatory effects of PS obtained in the individual experiments summarized in Figure 2A are plotted against the % of pre-constriction. Each dot corresponds to a single experiment. Linear fits are represented for AngII and PHE with 95% confident intervals. The parameters for the fit were 98.8 ± 8.9 (intercept), -2.4 ± 0.54 (slope), 0.54 ($R^2$), -0.74 (Pearson´s r) when AngII was used as vasoconstrictor and 35.03 ± 7.5 (intercept), -0.7 ± 0.24 (slope), 0.25 ($R^2$), -0.54 (Pearson´s r) in the case of PHE.

***Figure S6. Myography of renal arteries in response to TRPM3 activation.***

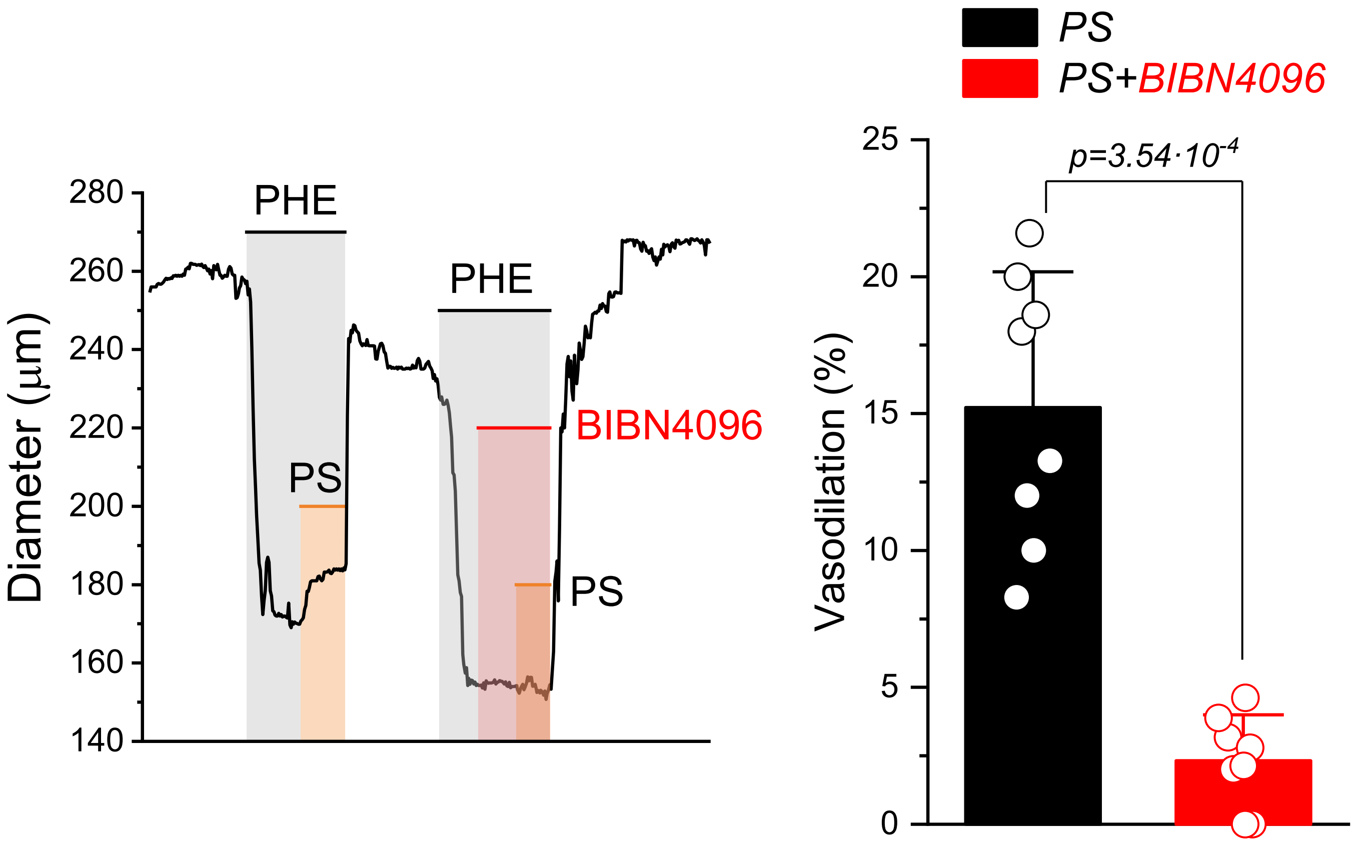


Left, representative experiment showing the vasodilation induced by PS (10 µM) under phenylephrine (Phe, 10 µM) stimulation before and after applying BIBN 4096 (1 µM) in a renal artery from a WT mice mounted in a pressure myograph. Average effects (mean ± SD) are shown in the bar graph in the right. (N = 8). Statistical significance was tested by paired Student's t-test.

***Figure S7. Effect of TRPM3 activation on renal flow after inhibiting COX pathway.***

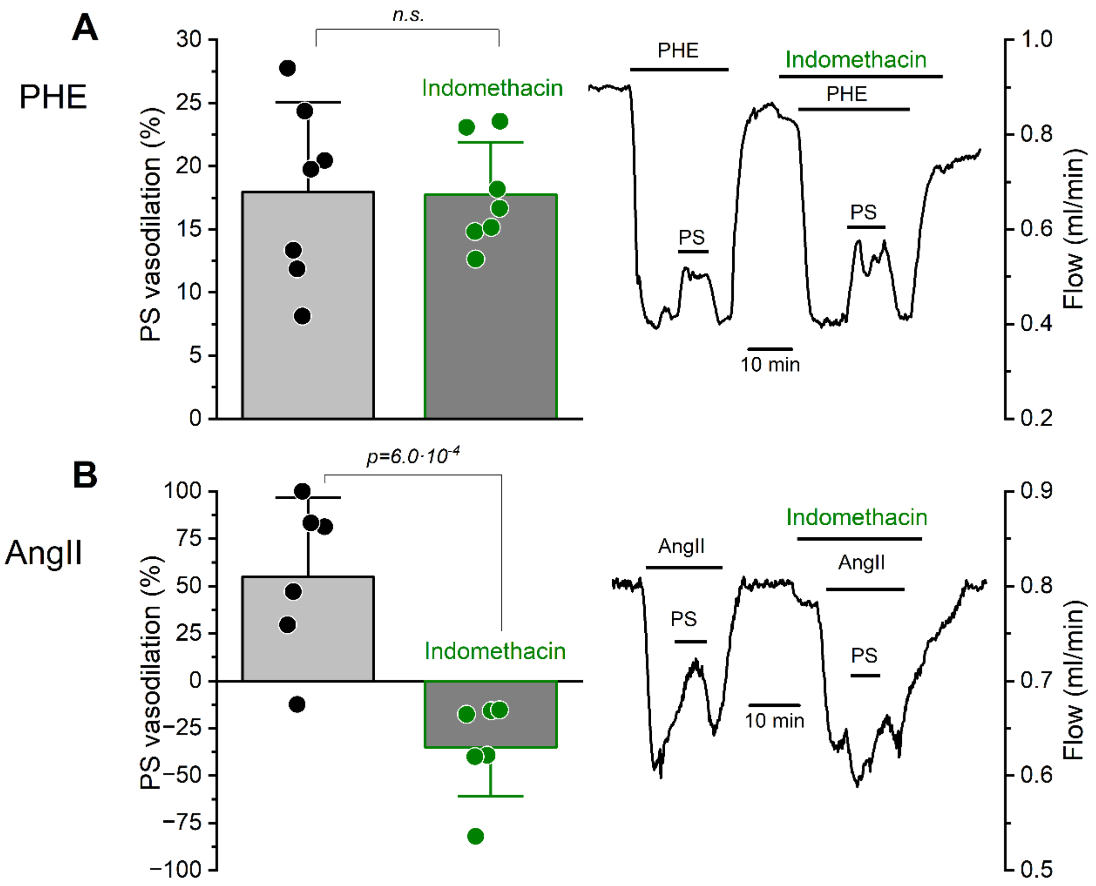


Responses to pregnenolone sulfate (PS, 10 μM) during phenylephrine (PHE, 1 μM; A) or angiotensin II (AngII, 0.5 nM; B)) induced vasoconstriction in isolated perfused kidneys from wild-type mice, in the presence or absence of indomethacin (10 μM) a blocker of COX. PS-induced vasodilation was unaffected by indomethacin treatment in PHE-precontracted renal arteries. In contrast, PS-induced vasodilation turns into vasoconstriction in the presence of indomethacin in AngII-preconstricted arteries Each bar is mean ± SD of 6-7 isolated perfused kidneys from different animals. Statistical significance was evaluated using Student’s paired t-test.

***Figure S8. β-gal expression and activity in kidney sections from WT and Trpm3-KO mice.***

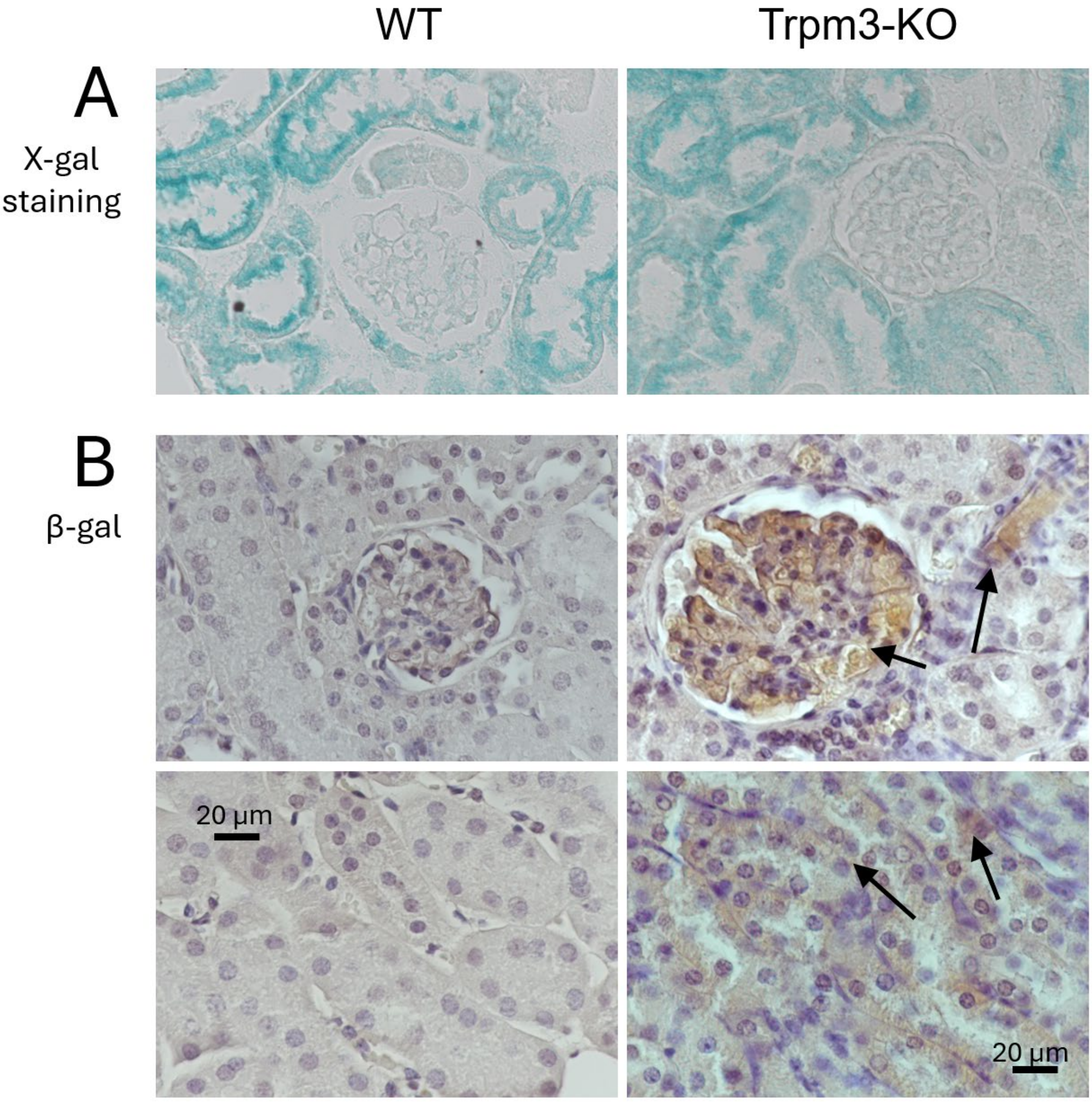


(A) X-gal staining of 5 µm kidney sections from wild-type (WT, left panel) or *Trpm3-KO* mice (right panel) showing β-galactosidase activity (blue staining) in both strains. (B) Representative microphotographs of immunohistochemistry using a recombinant β-galactosidase antibody revealed with DAB (brown labelling). Two different kidney sections from WT (left panels) and *Trpm3-KO* mice (right panels) showing regions with glomerulus or with tubular structures are displayed in each case. Specific labelling could be found only in *Trpm3-KO* sections, being clear in glomerulus. However, only low-intensity staining without a well-defined local distribution was observed in renal tubules. Gill's haematoxylin staining was used for nuclei. Each image is representative of at least 5 fields of 3-5 sections from 3-5 kidney samples from different male animals.

***Figure S9. Control experiments for RNAscope technique.***

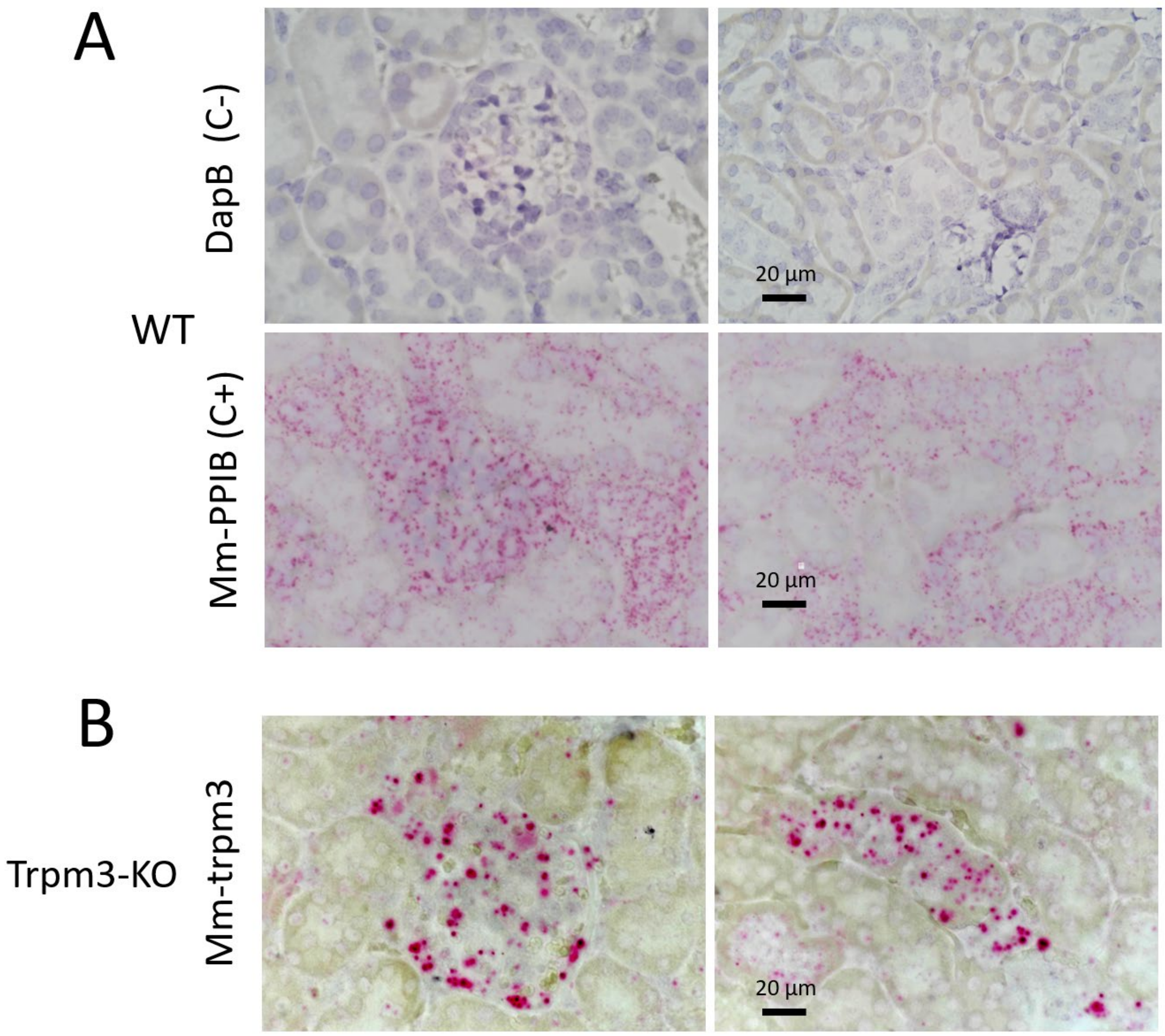


Representative brightfield images of 5 µm kidney sections from WT (A) or *Trpm3-KO* mice (B) are shown. In all cases, left panels show regions with glomeruli and right panels kidney sections with tubular structures The sections were stained with Gill's haematoxylin to visualize nuclei after hybridization with specific RNAscope® probes: Negative Control Probe DapB (panel A, "C-"), Positive Control Probe Mn-PPIB (panel B, "C+"), and Mm-Trpm3 mRNA probe (panel C). RNAscope in situ hybridization signals are indicated by red dots. Positive RNAscope signal is clearly observed in the *Trpm3-KO* Mm-Trpm3 mRNA probe, consistent with the previously described expression of a truncated protein in the KO mice. Images are obtained with a 40X objective. Each image is representative of at least 5 fields of 3-5 sections from 3-5 kidney samples from different male animals.

***Figure S10. TRPM3 mRNA localization in renal vessels and sympathetic nerves.***

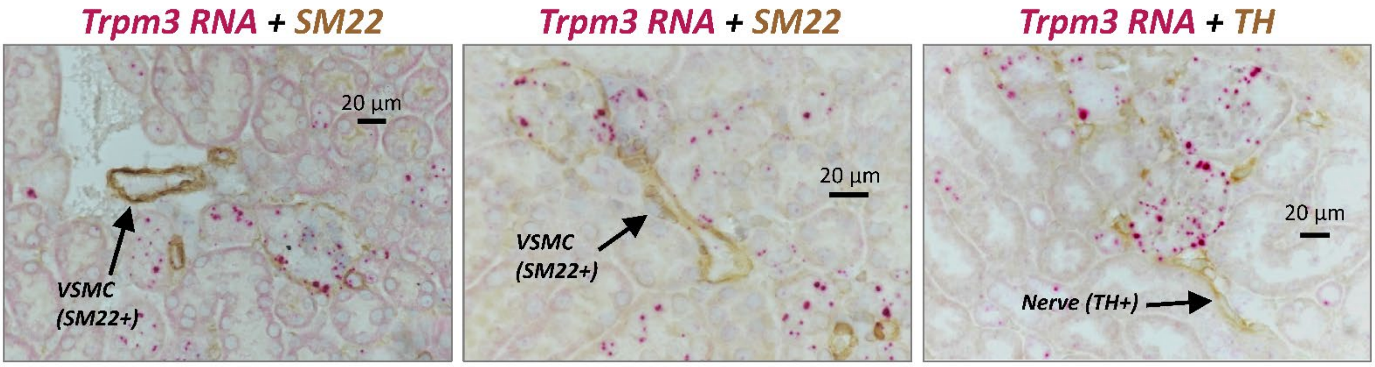


Brightfield images of 5 µm kidney sections from wild-type mice. Renal arteriole (left) and afferent arteriole (center) were identified by immunohistochemical staining for vascular smooth muscle cells (SM22, brown). Sympathetic nerve fibers (right) were identified by tyrosine hydroxylase (TH, brown). TRPM3 mRNA expression was detected by RNAscope in situ hybridization (red dots). Each image is representative of at least 5-8 areas, obtained from 2-5 sections from 4 different WT males.

# Tables

| | PHE | | AngII | |
|---|---|---|---|---|
| | WT | Trpm3-KO | WT | Trpm3-KO |
| **$E_{max}$ (%)** | 92.2 ± 17.7 | 79.7 ± 8 | 91.8 ± 6.3 | 75.9 ± 8.7 |
| **$EC_{50}$ (M)** | $9.5 \cdot 10^{-7} \pm 4.2 \cdot 10^{-7}$ | $7.1 \cdot 10^{-7} \pm 1.2 \cdot 10^{-7}$ | $17 \cdot 10^{-10} \pm 5 \cdot 10^{-10}$ | $8.6 \cdot 10^{-10} \pm 3.8 \cdot 10^{-10}$ |
| **n** | 1.1 ± 0.4 | 1.6 ± 0.45 | 0.7 ± 0.1 | 0.8 ± 0.2 |
| *R-square* | 0.71 | 0.83 | 0.87 | 0.893 |
| *F-test* | *F(3,78)=0.178, p=0.91* | | *F(3,87)=0.723, p=0.54* | |

**Table S1. Fit parameters of Figure 2A**

<table>
<tr><td></td><td colspan="4">AngII</td></tr>
<tr><td></td><td>WT</td><td>WT-Losartan</td><td>Trpm3-KO</td><td>Trpm3-KO-Losartan</td></tr>
<tr><td>$E_{max}$ (%)</td><td>94.1 ± 4.8</td><td>76.2 ± 4.2</td><td>81.9 ± 4.2</td><td>43.9 ± 3.08</td></tr>
<tr><td>$EC_{50}$ (M)</td><td>$15.8 \cdot 10^{-10} \pm 4.4 \cdot 10^{-10}$</td><td>$13.9 \cdot 10^{-10} \pm 3.2 \cdot 10^{-10}$</td><td>$11.7 \cdot 10^{-10} \pm 2.7 \cdot 10^{-10}$</td><td>$6.3 \cdot 10^{-10} \pm 2.4 \cdot 10^{-10}$</td></tr>
<tr><td>n</td><td>0.62 ± 0.08</td><td>0.84 ± 0.13</td><td>0.78 ± 0.1</td><td>0.77 ± 0.2</td></tr>
<tr><td>R-square</td><td>0.96</td><td>0.89</td><td>0.91</td><td>0.76</td></tr>
<tr><td rowspan="2">F-test</td><td colspan="2">F(3,52)=4.586, p=0.0063</td><td colspan="2">F(3,74)=29.753, p<0.0001</td></tr>
<tr><td></td><td colspan="3">F(3,71)=22.4108, p<0.0001</td></tr>
</table>

**Table S2. Fit parameters of Figure 2C**

$$E = \frac{E_{max}[Agonist]^n}{{EC_{50}}^n + [Agonist]^n}$$
